# Gut taste receptor type 1 member 3 is an intrinsic regulator of Western diet-induced intestinal inflammation

**DOI:** 10.1186/s12916-023-02848-0

**Published:** 2023-04-28

**Authors:** Woo-Jeong Shon, Jae Won Song, Seung Hoon Oh, Keon-Hee Lee, Hobin Seong, Hyun Ju You, Je Kyung Seong, Dong-Mi Shin

**Affiliations:** 1 Department of Food and Nutrition, Seoul National University College of Human Ecology, Gwanak-Gu, Seoul, 08826 Republic of Korea; 2 Research Institute of Human Ecology, Seoul National University, Seoul, Republic of Korea; 3 Bio-MAX/N-Bio, Seoul National University, Seoul, Republic of Korea; 4 Research Institute for Veterinary Science, College of Veterinary Medicine, Seoul National University, Seoul, Republic of Korea; 5 Korea Mouse Phenotyping Center, Seoul, Republic of Korea

**Keywords:** Butyrate-producing microbes, Clostridia, Gut-taste receptor, Inflammatory bowel disease, Intestinal PPAR-γ, mTOR signaling, Taste receptor type 1 member 3, Western diet

## Abstract

**Background:**

Long-term intake of a Western diet (WD), characterized by a high-fat content and sugary drinks, is hypothesized to contribute to the development of inflammatory bowel disease (IBD). Despite the identified clinical association, the molecular mechanisms by which dietary changes contribute to IBD development remain unknown. Therefore, we examined the influence of long-term intake of a WD on intestinal inflammation and the mechanisms by which WD intake affects IBD development.

**Methods:**

Mice fed normal diet or WD for 10 weeks, and bowel inflammation was evaluated through pathohistological and infiltrated inflammatory cell assessments. To understand the role of intestinal taste receptor type 1 member 3 (TAS1R3) in WD-induced intestinal inflammation, cultured enteroendocrine cells harboring TAS1R3, subjected to RNA interference or antagonist treatment, and *Tas1r3*-deficient mice were used. RNA-sequencing, flow cytometry, 16S metagenomic sequencing, and bioinformatics analyses were performed to examine the involved mechanisms. To demonstrate their clinical relevance, intestinal biopsies from patients with IBD and mice with dextran sulfate sodium-induced colitis were analyzed.

**Results:**

Our study revealed for the first time that intestinal TAS1R3 is a critical mediator of WD-induced intestinal inflammation. WD-fed mice showed marked TAS1R3 overexpression with hallmarks of serious bowel inflammation. Conversely, mice lacking TAS1R3 failed to exhibit inflammatory responses to WD. Mechanistically, intestinal transcriptome analysis revealed that *Tas1r3* deficiency suppressed mTOR signaling, significantly increasing the expression of PPARγ (a major mucosal defense enhancer) and upregulating the expression of PPARγ target-gene (tight junction protein and antimicrobial peptide). The gut microbiota of *Tas1r3*-deficient mice showed expansion of butyrate-producing Clostridia. Moreover, an increased expression of host PPARγ-signaling pathway proteins was positively correlated with butyrate-producing microbes, suggesting that intestinal TAS1R3 regulates the relationship between host metabolism and gut microflora in response to dietary factors. In cultured intestinal cells, regulation of the TAS1R3–mTOR–PPARγ axis was critical for triggering an inflammatory response via proinflammatory cytokine production and secretion. Abnormal regulation of the axis was observed in patients with IBD.

**Conclusions:**

Our findings suggest that the TAS1R3–mTOR–PPARγ axis in the gut links Western diet consumption with intestinal inflammation and is a potential therapeutic target for IBD.

**Supplementary Information:**

The online version contains supplementary material available at 10.1186/s12916-023-02848-0.

## Background

Inflammatory bowel disease (IBD) is a chronic, relapsing disorder of the gastrointestinal tract (GI); Crohn’s disease and ulcerative colitis are common forms of IBD [1]. Although the etiology of IBD remains unclear, it is believed to involve complex interactions among multiple genetic factors, altered gut microbiota, inappropriate host immune responses, and environmental factors [2, 3]. Traditionally, IBD has been more common in individuals of the Western European descent [4]. The majority of the > 215 genes with variants associated with an increased risk of IBD, including *NOD2* and *ATG16L*, have been identified in cohorts of Western patients with IBD [5], but not in Asian cohorts [6, 7]. Moreover, immigrants to Western countries have an increased risk of IBD [8, 9], suggesting that the Western environment and lifestyle contribute to the IBD pathogenesis. Both murine and human studies showed that intake of a Western diet (WD), characterized by a high-fat and sugar content, influences IBD pathogenesis [10–12]. Dietary surveys revealed that some patients with IBD believe that WD intake triggers disease flare-ups and exacerbates symptom severity [13, 14]. However, the precise molecular mechanisms underlying these phenomena are unknown.

Taste receptors (TRs) include two families of G protein-coupled receptors: taste receptor type 1 (T1R) and taste receptor type 2 [15]. The T1R family senses sweet (TAS1R2, TAS1R3) and umami (TAS1R1, TAS1R3) stimuli, whereas the taste receptor type 2 family senses bitter stimuli. TRs previously characterized in the oral cavity have recently been reported in extra-oral tissues, with particularly high expression in enteroendocrine cells (EECs) of the gastrointestinal tract [16]. TRs regulate the release of intestinal peptide hormones, including glucagon-like peptide 1 (GLP-1) [17]. GLP-1, secreted by EECs, potentiates glucose-induced insulin response, promotes beta-cell survival, slows gastric emptying, and regulates energy expenditure [18]. Although the mechanisms underlying TR function in the contexts of digestion and metabolism are well-characterized, their roles in intestinal inflammation and immunity are unclear.

Recent studies showed that taste chemosensory signaling proteins are expressed in intestinal tuft cells, where they play critical roles in detecting and eliminating intestinal parasites by initiating type II immune responses and regulating gut epithelial homeostasis [19–21]. These studies suggest that taste or taste-like chemosensory pathways in the intestine play important roles in regulating immune responses to components of luminal stimuli. However, most studies have primarily focused on elucidating the immunomodulatory reactions of these pathways in response to microorganisms or microbial metabolites. To our knowledge, no studies have reported the role of TR responses to endogenous dietary ligands. Given the immense diversity of non-microbial signals (nutrients and non-nutritive chemicals) that stimulate immune responses at the barrier site, intestinal TRs, which are directly activated by dietary ligands, may modulate intestinal mucosal immunity and inflammation.

In this study, we developed a murine model of severe intestinal inflammation triggered by long-term WD intake to evaluate the influence of WD intake on intestinal inflammation and investigate the mechanisms by which WD intake influence the development of IBD. We hypothesized that modulation of the nutrient-induced gut-taste receptor TAS1R3 (TR type 1 member 3), is central to the regulation of intestinal inflammation. Our findings provide novel insights into the previously unknown effects of long-term WD consumption on intestinal inflammation and may lead to the development of preventive or therapeutic strategies for IBD.

## Methods

### Study design

The objective of the study was to evaluate the influence of long-term WD intake on intestinal inflammation and investigate possible mechanisms by which WD intake could affect IBD development. To this end, mice were fed normal diet (ND) or WD for 10 weeks, and bowel inflammation was evaluated through pathohistological and infiltrated inflammatory cell assessments. To identify the mechanisms by which intestinal inflammation is prompted by WD, RNA-seq was performed on the inflamed intestinal tissues. Because the results revealed increased expression of the nutrient-sensing taste receptor TAS1R3 in inflamed bowel tissues, we hypothesized that nutrient-induced TAS1R3 modulation is central to regulating intestinal inflammation. We first determined the dietary ligand(s) responsible for strongly activating TAS1R3 using in vitro assessment of TAS1R3-expressing enteroendocrine cells (EECs) to confirm whether TAS1R3 could trigger intestinal inflammation, and then assessed the downstream molecular changes. To understand the role of TAS1R3 in WD-induced intestinal inflammation, we investigated changes in intestinal gene expression profiles, inflammatory cell infiltration in intestinal tissues, and the gut microbiome of *Tas1r3*-deficient and littermate wild-type mice fed WD. Finally, we confirmed the expression of TAS1R3 and downstream players in intestinal biopsies of patients with IBD ex vivo to demonstrate their relevance to disease. All mice were assigned randomly to treatment groups, and animals demonstrating sickness or severe stress were euthanized and excluded. Otherwise, all data were included in the study. The experimental protocol was approved by the Seoul National University Institutional Animal Care and Usage Committee (approval SNU-181001-2).

### Mice

C57BL/6 (JAX 000664) and Tas1r3tm1Csz (JAX 013066) mice were obtained from the Jackson Laboratory (West Grove, PA, USA). *Tas1r3*-knockout (*Tas1r3*^−/−^) mice were backcrossed to C57BL/6 mice for at least seven generations. The animals were genotyped using standard PCR. The mice (5–6 weeks old; 23–25 g) were randomly assigned to groups. Age- and weight-matched male and female littermates were used as controls. *Tas1r3*^−/−^ and wild-type littermate control (*Tas1r3*^+/+^) mice were housed under constant temperature (23 ± 2 °C) and humidity (55–60%) conditions in a specific pathogen-free animal facility. All mice were housed in the same room to minimize environmental effects.

### Diets

To imitate the human WD, characterized by a high-fat content and sugary drinks, mice were fed high-fat diet and sucrose solution. The high-fat diet (60%; D12492) and matching normal diet (ND; D12450J) pellets were purchased from Research Diets (New Brunswick, NJ, USA). The sugar concentration in the sucrose solution was determined, as described in a previous rodent study [22]. Two independent series of experiments were conducted, and mice were randomly assigned as follows:

In experiment 1, two groups of mice were used: (i) ND—mice administered ND with plain water (control group; *n* = 10 mice) and (ii) WD—mice administered the high-fat diet with sucrose solution (*n* = 10 mice).

In experiment 2, four groups of mice were used: (i) *Tas1r3*^+/+^ mice who were administered ND (*n* = 10 mice), (ii) *Tas1r3*^−/−^ mice who were administered ND (*n* = 10 mice), (iii) *Tas1r3*^+/+^ mice who were administered WD (*n* = 10 mice), and (iv) *Tas1r3*^−/−^ mice who were administered WD (*n* = 10 mice). Food and water were supplied ad libitum for 10 weeks. The body weight, as well as food and water intake, were monitored weekly.

All mice used in the experiments were randomly assigned to each group, and random numbers generated using SPSS software (version 18.0; SPSS Inc., Chicago, IL, USA) were assigned with a unique code linking to the individual animal.

### Histology

After 10 weeks of diet induction or 7 days of 2% dextran sulfate sodium (molecular weight: 36,000–50,000 kDa; Cat# 02160110-CF; MP Biomedicals, Santa Ana, CA, USA) induction, all mice were anesthetized and euthanized by intraperitoneal injection of 20% urethane (U2500; Sigma-Aldrich, St. Louis, MO, USA), and the entire intestine was removed and opened longitudinally. Small and large intestinal tissues were fixed overnight in 10% formalin and embedded in paraffin. The tissue blocks were cut into Sects. (4–6 μm thick) that were mounted on glass slides, stained with hematoxylin and eosin, and photographed under a Nikon Eclipse TE2000-U microscope (Tokyo, Japan) equipped with a QImaging digital camera (Teledyne QImaging, Surrey, BC, Canada). H&E-stained intestinal sections were coded for blind microscopic assessment of inflammation. Sections coded for assessment of inflammation were scored by two blinded investigators as described previously (*n* = 10 mice/group) [23].

### Immunohistochemistry and immunofluorescence

Serial sections (4–6 μm thick) were prepared from formalin-fixed paraffin-embedded specimens and mounted on silane-coated slides (Dako Japan Co., Ltd., Kyoto, Japan). Tissues were deparaffinized and rehydrated through a graded xylene and alcohol series, placed in citrate-buffered solution (pH 6.0) (C9999; Sigma-Aldrich), and heated in a microwave oven to 100 °C for 20 min for antigen retrieval. After washing with phosphate-buffered saline, the slides were incubated with phosphate-buffered saline-Tween with 1% bovine serum albumin (Sigma-Aldrich) for 1 h, incubated overnight at 4 °C with anti-TAS1R3 antibodies (1:100) (Cat#OSR00184W, RRID: AB_2271552; Invitrogen, Carlsbad, CA, USA) or pan-leukocytes (CD45; 1:100) (Cat#ab10558, RRID: AB_442810; Abcam, Cambridge, UK), and developed using a Mouse and Rabbit-Specific HRP/DAB Detection IHC Kit (Cat#ab64264; Abcam), according to the manufacturer’s instructions. Primary antibodies used for small or large intestine immunofluorescence staining included mouse anti-CD4 (Cat#14–0041-86, RRID: AB_467065; eBioscience, San Diego, CA, USA), rat anti-CD8 (Cat#ab22378, RRID: AB_447033; Abcam), and mouse anti-CD11b (Cat#14–0112-82, RRID: AB_467108; eBioscience), to label different types of immune cells in intestinal tissues. Secondary and secondary-conjugated primary antibodies used for small intestine and colon immunofluorescence staining included Alexa Fluor 488 anti-mouse/human CD11b (Cat#101219, RRID: AB_493545; BioLegend, San Diego, CA, USA), Alexa Fluor 594 donkey anti-rabbit (Cat#A21207, RRID: AB_141637; Thermo Fisher Scientific, Waltham, MA, USA), Alexa Fluor 488 donkey anti-mouse (Cat#A21202, RRID: AB_141607; Thermo Fisher Scientific), and Alexa Fluor 594 donkey anti-rat (Cat#A21209, RRID: AB_2535795; Thermo Fisher Scientific). Immunoreactivity was visualized using a confocal microscope system (LSM 510; Carl Zeiss, Oberkochen, Germany). Images were captured at 20X magnification using an EVOS FL Cell Imaging System (Thermo Fisher Scientific) and a BZ-X710 All-in-One Fluorescence Microscope (Keyence, Osaka, Japan), and analyzed using ImageJ software (National Institutes of Health, Bethesda, MD, USA). Tissue-infiltrated cells are expressed as the ratio of the stained area to the total area of the measured tissue region (*n* = 10 mice/group).

### Enterocyte cell culture and GLP-1 secretion assay

Human enteroendocrine NCI-H716 cells (CCL-251; American Type Culture Collection, Manassas, VA, USA) were maintained in suspension culture at 37 °C and 5% CO_2_, according to the supplier’s protocol. The culture medium was RPMI-1640 (Invitrogen) supplemented with 10% fetal bovine serum, 2 mM L-glutamine, 100 IU/mL penicillin, and 100 μg/mL streptomycin. Two days prior to the experiments, 1 × 10^6^ cells were seeded in 24-well culture plates precoated with Matrigel (BD Biosciences, Franklin Lakes, NJ, USA), as described previously [24]. On the day of the experiments, the supernatants were replaced with medium containing 10 mM glucose (Cat# G7021), 10 mM fructose (Cat# F3510), 10 mM sucrose (glucose + fructose), and/or 10 μM palmitate (Cat# P5585). In the TAS1R3 antagonist experiment, NCI-H716 cells were pretreated with the TAS1R3 antagonist, lactisole (2.5 mM) (Cat# M6546) for 30 min and stimulated with medium containing fructose (10 mM), glucose (10 mM), and palmitate (10 μM) for 12 h. In the PPARγ antagonist experiment, NCI-H716 cells were transfected with *TAS1R3* siRNA or control siRNA for 48 h, and then stimulated with the PPARγ antagonist, GW9662 (Cat# M6191) (10 μM), in the presence of fructose (10 mM), glucose (10 mM), and palmitate (10 μM) for 12 h. The cells were incubated for 2, 4, 12, and 24 h at 37 °C with or without different test agents and inhibitors. Glucose, fructose, palmitate, lactisole, and GW9662 were purchased from Sigma-Aldrich. Following incubation, the medium was collected, centrifuged at 1,000 × *g* for 10 min at 4 °C, to remove any floating cells, and frozen at -20 °C for subsequent biochemical analysis. GLP-1 was measured using a commercial GLP-1 (active) Enzyme-Linked Immunosorbent Assay Kit (Cat#EGLP-35 K; Millipore, Billerica, MA, USA), according to the manufacturer’s protocol (*n* = 3/group).

### siRNA knockdown

siRNA duplexes for *TAS1R3* were synthesized by Bioneer (Daejeon, South Korea). Scrambled negative control siRNA was also purchased from Bioneer. For knockdown experiments, 5 × 10^5^ endocrine differentiated NCI-H716 cells were plated into 6-well plates and cultured for 48 h. *TAS1R3* (10 nM) or control (10 nM) siRNA was transfected into the cells using Lipofectamine RNAiMAX Reagent (Invitrogen). After 48 h of transfection, the cells were induced with medium containing 10 mM glucose, 10 mM fructose, and 10 μM palmitate for 12 h (*n* = 3/group). The target sequences of the siRNA are listed in Additional file 1 (Table S1).

### Enzyme-linked immunosorbent assay

Enzyme-Linked Immunosorbent Assay (ELISA) Kits specific for human TNF-α (Cat# ab181421) and IL-8 (Cat# ab46032) were purchased from Abcam. TNF-α and IL-8 in the cell-conditioned medium were quantified using Enzyme-Linked Immunosorbent Assay, according to the manufacturer’s instructions. Briefly, the samples were diluted with assay buffer and added to microwells precoated with anti-human TNF-α or IL-8 antibodies, followed by incubation with an antibody cocktail and 3,3′,5,5′-tetramethylbenzidine substrate. Conjugated enzyme activity was detected by measuring absorbance at 450 nm (*n* = 3/group).

### RNA isolation and quantitative reverse transcription-PCR

Total RNA was extracted from the ileum tissue of WD- or DSS-induced wild-type and knockout mice and NCI-H716 cells. After harvest, the ileal and cell extracts were immediately snap-frozen by immersion in liquid nitrogen and stored at –80 °C until RNA extraction. Total RNA was isolated using RNAqueous (Cat# AM1914; Ambion, Austin, TX, USA), according to the manufacturer’s instructions. RNA extraction involved a DNase treatment step. RNA was quantified using a NanoDrop 2000/2000c Spectrophotometer (Thermo Fisher Scientific), and 1 μg of RNA from each sample was used for cDNA synthesis (Cat# 11917010; Invitrogen). Quantitative reverse transcription-PCR was performed using StepOnePlus (Real-time PCR System; Applied Biosystems, Foster City, CA, USA) and SYBRGreen (Cat# 4367659; Applied Biosystems). The relative quantification of gene expression was performed using the 2^−△△Ct^ method. The cycle threshold values of the genes of interest were normalized to that of *Gapdh* (*n* = 3–10 mice/group). The primers used for quantitative reverse-transcription-PCR are listed in Additional file 1 (Table S2).

### RNA-sequencing (RNA-seq)

Isolated RNA (1000 ng) was used as input for library generation. Intact mRNA was isolated from total RNA using a Dynabeads mRNA DIRECT Micro Kit (Ambion). The total mRNA samples were depleted of up to 99.9% of 5S, 5.8S, 18S, and 28S rRNA using the RiboMinus Eukaryote System v2 (Life Technologies, Carlsbad, CA, USA). Barcoded cDNA libraries were prepared from the rRNA-depleted mRNA samples and constructed using the Ion Total RNA Seq Kit v2 (Life Technologies). Whole-transcriptome libraries were diluted to 100 pM and amplified with ion sphere particles by emulsion PCR using an Ion One Touch 2 System (Life Technologies) and Ion PI Hi-Q OT2 200 Kit (Cat# A26434; Life Technologies). Template-positive ion sphere particles were enriched using the Ion OneTouch Enrichment System (Life Technologies), in which biotinylated adaptor sequences were selected by binding to streptavidin-conjugated beads. The template-positive ion sphere particles were sequenced using the Ion PI Hi-Q Sequencing 200 Kit (Cat# A26433; Life Technologies). Sequencing primers were annealed to template fragments attached to the ion sphere particles, and the template-positive ion sphere particle samples were loaded onto a chip from the Ion PI Chip Kit v3 (Cat# A26771; Life Technologies) and incubated with polymerase. Finally, the chip was placed on an Ion Proton System (Life Technologies) for ion semiconductor sequencing, which is based on the principle of hydrogen ion release detection when nucleotides are incorporated into the growing DNA template (*n* = 6 mice/group). All procedures were performed according to the manufacturer’s instructions.

### Bioinformatics analysis of RNA-seq data

Raw RNA-seq reads were split into individual samples based on their barcodes and quality controlled using the FASTQC tool. The reads were analyzed using Partek Flow software (Partek, St. Louis, MO, USA) [25]. Briefly, the reads were mapped to Genome Reference Consortium Mouse Build 38 (mm10) using STAR 2.5.3a aligner. Quantification was performed using the transcript model Ensembl Transcripts release 91. Differential expression analysis was conducted using R package DESeq2. Genes with an absolute fold-change of ≥ 2 and a false discovery rate (FDR) < 0.01 were considered as differentially expressed genes (DEGs). The DEGs were subjected to ingenuity pathway analysis using IPA software (Qiagen, Hilden, Germany) [26]. Hierarchical clustering and biological classification analyses were also performed. Fisher’s exact test was used to determine the significance of the enrichment of specific biological processes among the DEGs. Hierarchical clustering analysis was performed using Genesis v1.7.545 based on a Pearson correlation distance matrix and average linkage algorithm.

### Protein extraction and western blotting

Approximately 0.1 g of terminal ileum tissue isolated from *Tas1r3*^+/+^ or *Tas1r3*^−/−^ mice was transferred to 1 mL Tissue Extraction Reagent 1 (Cat# FNN0071; Invitrogen) with 1/1,000 protease inhibitor (Cat# P-2714; Sigma-Aldrich, St. Louis, MO, USA) and homogenized. NCI-H716 cells were also lysed using RIPA buffer and homogenized. Protein concentrations were determined using the Pierce BCA Protein Assay Kit (Cat# 23227; Thermo Fisher Scientific) with bovine serum albumin as the standard. Aliquots of each protein lysate (20 μg) were subjected to sodium dodecyl sulfate polyacrylamide gel electrophoresis, transferred to a polyvinylidene fluoride membrane, blocked for 1 h at 24 °C with 5% fat-free milk in Tris-buffered saline containing 0.1% Tween 20, and incubated with monoclonal rabbit anti-mTOR (1:1,000) (Cat# 2972S, RRID: AB_330978), rabbit anti-phospho-mTOR (1:1,000) (Cat# 2971S, RRID: AB_330970), and rabbit anti-PPARgamma (1:1,000) (Cat# 2443S, RRID: AB_823598) primary antibodies from Cell Signaling Technology (Danvers, MA, USA), and rabbit anti-Occludin (1:2,000) (Cat# ab216327, RRID:AB_2737295) and rabbit anti-Claudin-1 (1:2,000) (Cat# ab180158) primary antibodies from Abcam. Mouse anti-α-tubulin (1:10,000) (Cat# T5168, RRID: AB_477579) from Sigma was used as a control. Horseradish peroxidase-conjugated goat anti-rabbit secondary antibodies (1:5,000) (Cat# 7074S, RRID: AB_2099233) from Cell Signaling Technology and goat anti-mouse secondary antibodies (1:10,000) (Cat# G21040, RRID: AB_2536527) from Invitrogen were used for detection. The target proteins were detected using enhanced chemiluminescence western blot detection reagents (Amersham Pharmacia Biotech, Piscataway, NJ, USA) (*n* = 6 mice/group).

### 16S rRNA gene sequencing

Fecal samples were frozen immediately after collection and stored at –80 °C. Total bacterial DNA was isolated from fecal samples using a QIAamp Fast DNA Stool Mini Kit (Cat# 51604; Qiagen), according to the manufacturer’s protocol for pathogen detection, with slight modifications. Briefly, approximately 200 mg of fecal material was homogenized for 1 min at 30 Hz with 5-mm sterilized steel beads in ASL buffer using a TissueLyser bead mill (Qiagen). The suspension was heated at 95 °C to lyse gram-positive bacterial cells. In the final incubation step, we extended the elution time from 1 to 5 min to increase the DNA yield. A 16S rRNA sequencing library was constructed, according to the 16S metagenomics sequencing library preparation protocol (Illumina, San Diego, CA, USA), targeting the V3 and V4 hypervariable regions of the 16S rRNA gene. KAPA HiFi HotStart ReadyMix (KAPA Biosystems, Wilmington, MA, USA) and the Agencourt AMPure XP System (Beckman Coulter Genomics, Brea, CA, USA) were used to amplify and purify the PCR products, respectively. The amplicons were sequenced in paired-end mode (PE275) using a MiSeq System (Illumina) (*n* = 10–14 mice/group).

### 16S rRNA-seq analysis

Paired sequences were dereplicated using the QIIME pipeline [27], and de novo and reference-based chimeras were removed using UCHIME software. Sequences from all samples were merged and sorted based on their relative abundances, and then closed operational taxonomic unit selection was performed using a 99% similarity threshold, followed by stringent taxonomic assignment using Silva v1.19. Based on the operational taxonomic unit abundance matrix and respective taxonomic classifications, feature abundance matrices were calculated at different taxonomic levels (from genus to phylum). To estimate the species diversity within samples (α-diversity), Shannon’s and Pielou’s indices were calculated using the R package phyloseq, after rarefaction. For comparisons among samples (β-diversity), Bray–Curtis and weighted principal coordinate analysis (PCoA) UniFrac dissimilarities were determined using phyloseq, based on profiles normalized to sample depth. Linear discriminant analysis effect size analysis was conducted online using the Galaxy workflow framework [28] to identify differentially abundant bacterial genera.

### Stool butyrate assay

Aliquots (200 mg) of fecal samples were thawed, to which 4 volumes of distilled water (800 μL) were added, followed by vortexing at room temperature for 5 min until the samples were homogenized. Then, 15 μL of 95% sulfuric acid (Sigma-Aldrich) was added to 300 μL of fecal supernatant for acidification (final 5% (v/v)), followed by stabilization for 5 min. After centrifugation at 14,000 × *g* for 5 min, the supernatants were transferred to a new tube. To extract volatile materials, 30 μL of 1% (v/v) internal standard (2-methylpentanoic acid; Sigma-Aldrich) and 300 μL of anhydrous ethyl ether (Sigma-Aldrich) were added to acidified fecal supernatant. The samples were vortexed for 1 min, and then centrifuged at 14,000 × *g* for 5 min. The upper layer was carefully transferred to a GC vial and stored at -80 °C before analysis. The 10 mM butyrate solution (Cat# T8626; Sigma-Aldrich) was used as the standard for butyrate analysis. Stool butyrate was measured using the Agilent Technologies 7890A GC System (Santa Clara, CA, USA), as described by David et al. [29] (*n* = 20 mice/group).

### IBD gene expression profiling

To analyze the DEGs in IBD (*n* = 204) *versus* non-IBD (*n* = 74), mRNA microarray expression profiles were retrieved and downloaded from the Gene Expression Omnibus database [30] by searching the following keywords: “IBD,” “active ulcerative colitis,” “active Crohn’s disease,” and “*Homo sapiens*” (organism). The inclusion criteria were as follows: (i) intestinal tissues (not cells) from adult patients with active IBD and (ii) samples from patients with IBD who had not received any interventions or treatments. After screening, five mRNA expression datasets (GSE160804, GSE126124, GSE95095, GSE75214, and GSE53306) were selected for analysis (see Additional file 1: Table S3). The raw microarray data were downloaded from the Gene Expression Omnibus database and preprocessed using Partek Genomics Suite version 6.6 (Partek) with the robust multichip analysis algorithm, which performs background adjustment, quantile normalization, and probe summarization. GC-content correction was used, as suggested by the default pipeline of Partek Genomics Suite. To estimate the effect of the normalization procedure, expression data without normalization and with standard robust multichip analysis normalization (without GC-content correction) were also generated. Differential gene expression analysis was performed using R/Bioconductor [31, 32]. For DEG selection, an FDR *P* < 0.001 was considered the cutoff value.

### Murine colitis model

Colitis was induced by administering 2% DSS dissolved in drinking water for 7 days. Control mice were provided with drinking water without DSS. Colitis development was evaluated by monitoring daily weight changes. Colitis severity was also scored by evaluating the clinical disease activity through daily observation of the following parameters: weight loss (0 points = no weight loss or weight gain, 1 point = 5–10% weight loss, 2 points = 11–15% weight loss, 3 points = 16–20% weight loss, 4 points =  > 21% weight loss); stool consistency (0 points = normal and well-formed, 2 points = very soft and unformed, 4 points = watery stool); and bleeding stool score (0 points = normal color stool, 2 points = reddish color stool, 4 points = bloody stool). The disease activity index was calculated based on the combined scores of weight loss, stool consistency, and bleeding, and ranged from 0 to 12. All parameters were scored from Days 0 to 7. On Day 7 after DSS-colitis induction, the mice were sacrificed, and the entire intestine was quickly removed. After determining the colon length as a marker of inflammation, the entire colon was cut open lengthways and gently flushed with sterile phosphate-buffered saline to remove any traces of feces. Small and large intestinal segments were immediately frozen in liquid nitrogen and stored at − 80 °C for subsequent extraction of total RNA. For histological analysis, intestinal segments were fixed in 10% neutral buffered formalin phosphate and stored at room temperature until inflammation was analyzed (*n* = 7 mice/group).

### Statistical analysis

All data were analyzed using GraphPad Prism software (version 9.0; GraphPad Inc., San Diego, CA, USA). The Kolmogorov–Smirnov test was used to assess data normality. All data were found to be normally distributed. SigmaPlot 11.0 was used to estimate all sample sizes using data from previous experiments and preliminary data with α = 0.05 and β = 0.2. Unless otherwise specified, all data are expressed as means ± standard errors of the mean. Descriptive statistics are listed in Additional file 1 (Table S4). The mean values of two groups were compared using Student’s *t*-test, and the means of multiple groups were compared using one-way analysis of variance, followed by Bonferroni post-hoc test. All statistical analyses were two-sided, and *P* < 0.05 was considered significant. In gene expression analyses, significant enrichment of specific genes was determined using a right-tailed Fisher’s exact test. Correlations between the relative abundance of butyrate-producing bacteria and transcript expression of PPAR-associated molecules were analyzed using Spearman’s correlation test.

## Results

### TAS1R3 expression is significantly increased in WD-induced intestinal inflammation

**Fig. 1:**
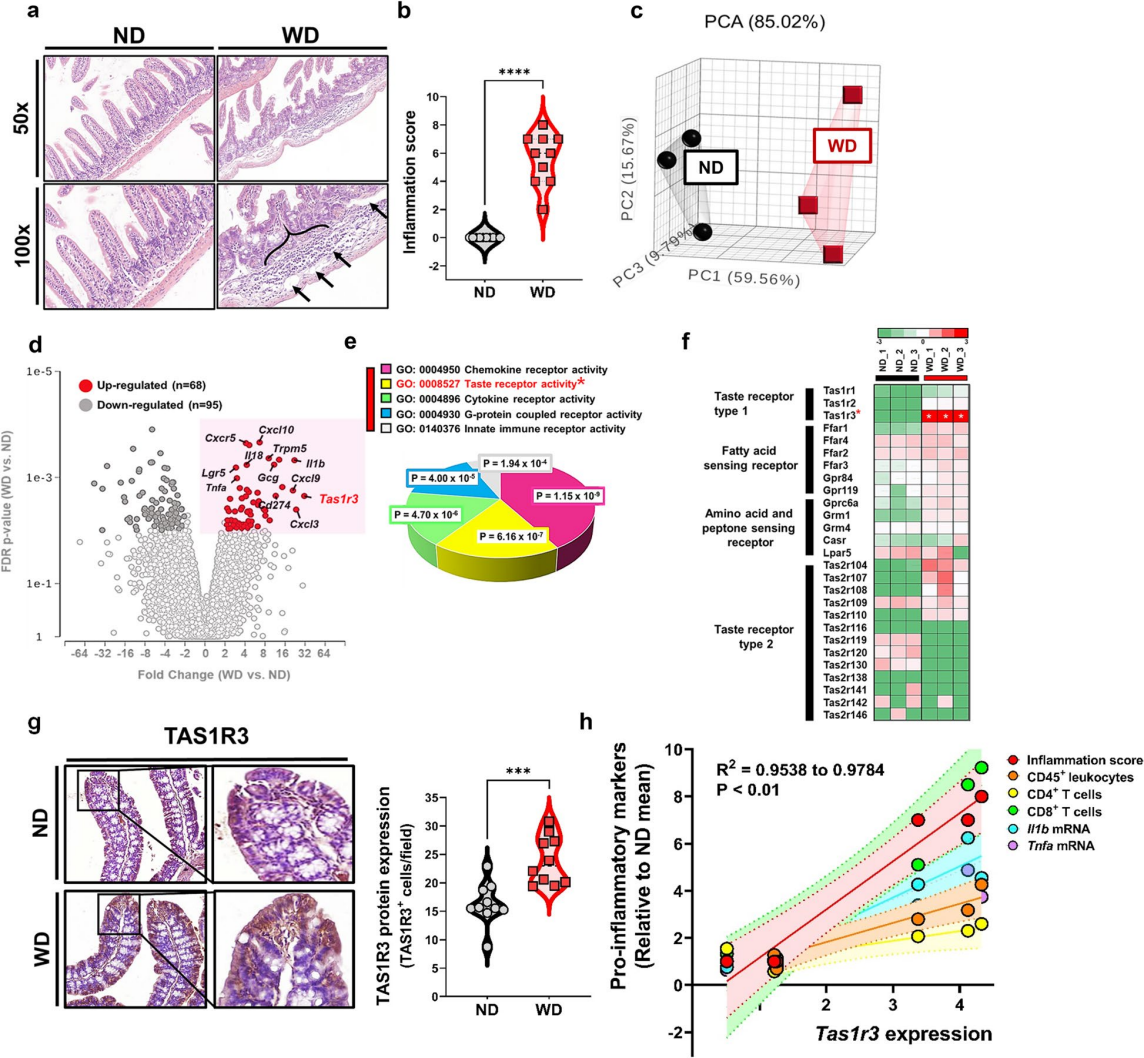
Increased TAS1R3 expression in WD-induced intestinal inflammation.** a** Representative hematoxylin and eosin-stained images of small intestine (terminal ileum) sections from mice fed ND (control) or WD (*n* = 10 mice/group). Edematous submucosa and severe cellular infiltration (arrows) (scale bars: 200 μm). **b** Histological scores (*n* = 10 mice/group). The sum of the severity scores for inflammation (0–3), damage (0–4), and extension (0–4). **c** PCA of the transcriptome of terminal ileum tissue. Points represent individual mice (*n* = 3 mice/group). **d** Volcano plot of differentially expressed genes between ND- and WD-fed mice. FDR *P*-values and fold-changes were obtained from RNA-sequencing data. **e** DAVID GO molecular function analysis of significantly upregulated genes. Significance (–log (*P*-value)) was calculated using a right-tailed Fisher’s exact test. **f** Heatmap of taste receptor activity-associated gene expression between groups. Data represent the average normalized intensities (*n* = 3 mice/group). The significance is indicated as a white * symbol in the heatmap. **g** Immunohistochemical staining of TAS1R3 in terminal ileum tissue (*n* = 10 mice/group). **h** Correlation between *Tas1r3* expression and pro-inflammatory markers (indicated cell type frequency and mRNA expression) in intestinal tissue (*n* = 3 mice/group). All values are relative to the ND mean (mean ± standard error). ***P < 0.001 and ****P < 0.0001 (unpaired Student’s *t*-test). FDR, false discovery rate; GO, gene ontology; ND, normal diet; PCA, principal component analysis; TAS1R3, taste receptor type 1 member 3; WD, Western diet

To investigate the effects of increased WD intake on intestinal inflammatory outcomes, C57BL/6 J mice were fed a high-fat diet and sugar water for 10 weeks and compared with control mice fed a ND and plain water. Bowel inflammation was markedly induced by long-term overconsumption of WD. Histopathological examination of the terminal ilea showed that WD induced severe tissue damage with significant cellular infiltration, extensive erosion, ulceration, and muscularis mucosa layer destruction (Fig. 1a, b).

To clarify the mechanisms by which WD induces intestinal inflammation, RNA-seq was performed on inflamed (WD-fed) and healthy (ND-fed) terminal ileum tissue. Principal component analysis (PCA) confirmed that the gene expression profiles of WD-fed mice were easily distinguished from those of ND controls (Fig. 1c). Transcriptome analysis revealed upregulation of pro-inflammatory cytokine- and chemokine-associated genes, including *Il1b*, *Il18*, *Tnfa*, *Cxcl3*, *Cxcl9*, and *Cxcl10*, in WD-fed mice. Strikingly, genes involved in chemosensory signaling pathways, including *Tas1r3*, *Trpm5*, *Lgr5*, and *Gcg*, were also expressed at high levels in WD-fed mice (Fig. 1d). Gene set enrichment analysis (GSEA) revealed taste receptor activity as the second-most overexpressed network in WD-induced bowel inflammation, following chemokine receptor activity (Fig. 1e). These findings indicate that long-term consumption of WD altered the expression of mRNA related not only to pro-inflammatory responses but also to receptor signaling pathways, including intestinal TRs.

Notably, *Tas1r3* mRNA expression (*P* < 0.0001; Fig. 1f) and protein abundance (*P* = 0.0004; Fig. 1g) were only significantly upregulated among the several G-protein-coupled TRs in WD-fed mice, but not in ND-fed mice. However, the expression of dimer-forming *Tas1r1* and *Tas1r2* did not differ between groups (Fig. 1f). *Tas1r3* expression was significantly and positively correlated with pro-inflammatory markers: histological inflammation score, proportion of CD45^+^ leukocytes (IFN-γ^+^TNF-α^+^CD4^+^ and CD8^+^ T cells) in draining mesenteric lymph nodes, as well as *Il1b* and *Tnfa* mRNA expression in ileal tissue (Fig. 1h). Collectively, these results show that significantly elevated TAS1R3 expression levels are a hallmark in WD-induced intestinal inflammation.

### A combination of glucose, fructose, and palmitate ligands upregulates pro-inflammatory cytokine and chemokine expression via TAS1R3 activation

To confirm whether TAS1R3 activation initiates an inflammatory response in the gut, we next studied cultured NCI-H716 EECs, an intestinal epithelial model with high TAS1R3 expression (see Additional file 2: Fig. S1a). Although sweet molecules are prominent TAS1R3 ligands [33], our in vivo findings showed that TAS1R3 expression was highest in the gut when dietary fat and sugar were combined. Accordingly, we used palmitate (the most abundant dietary fat) and/or sugars (glucose, fructose, and glucose + fructose) as ligands to determine whether TAS1R3 expression is increased by dietary fat in vitro.

**Fig. 2:**
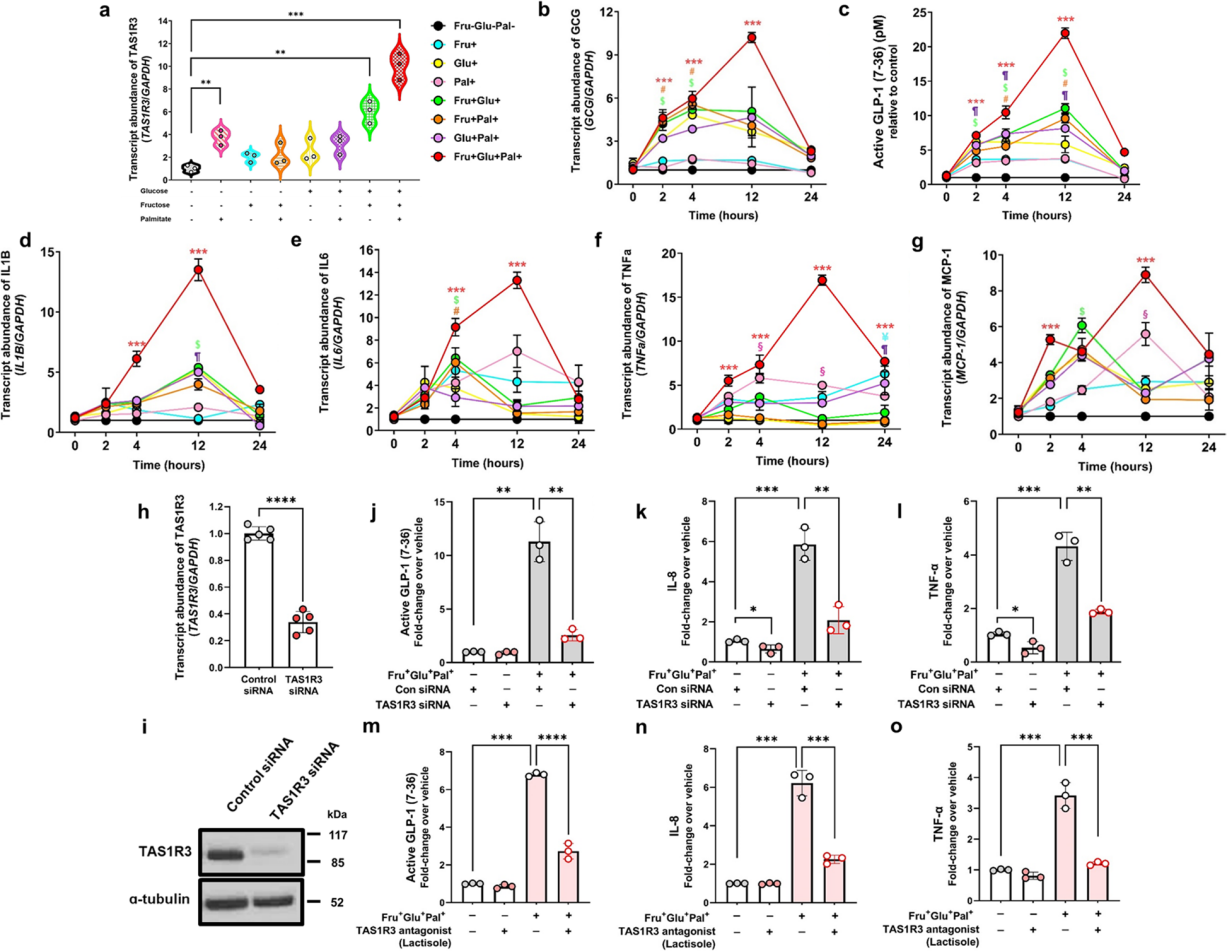
A combination of glucose, fructose, and palmitate ligands induces production and secretion of pro-inflammatory cytokines and chemokine in EECs via TAS1R3 activation.** a** qRT-PCR of relative mRNA expression of intestinal *TAS1R3* in NCI-H716 cells after 12 h of treatment with individual or combined glucose, fructose, and palmitate to activate TAS1R3 (*n* = 3/group). Time course of (**b**) *GLP1* mRNA expression, **c** active GLP-1 levels, **d** *IL1B* mRNA expression, **e** *IL6* mRNA expression, **f** *TNFɑ* mRNA expression, and **g** *MCP1* mRNA expression in NCI-H716 cells after stimulation with individual or combined glucose, fructose, and palmitate (*n* = 3/group). **h–l** NCI-H716 cells were transfected with TAS1R3 (10 nM) or scrambled control (10 nM) siRNA for 48 h and then stimulated with or without fructose (10 mM), glucose (10 mM), and palmitate (10 μM) for 12 h (*n* = 3/group). Evaluation of *TAS1R3* siRNA **h** mRNA and **i** protein knockdown in EECs. Transfection with *TAS1R3* siRNA inhibited *TAS1R3* mRNA and protein expression in differentiated NCI-H716 cells. The collected supernatants were assayed for active **j** GLP-1, **k** IL-8, and **l** TNF-α levels. **m–o** NCI-H716 cells were pretreated with the TAS1R3 antagonist, lactisole (2.5 mM), for 30 min and then stimulated with fructose (10 mM), glucose (10 mM), and palmitate (10 μM) for 12 h (*n* = 3/group). The collected supernatants were assayed by active **m** GLP-1, **n** IL-8, and **o** TNF-α enzyme-linked immunosorbent assay. Data represent means ± standard errors of the mean of three to five independent experiments. *P < 0.05, **P < 0.01, ***P < 0.001, and ****P < 0.0001 (one-way analysis of variance followed by Bonferroni post-hoc test). EEC, enteroendocrine cell; GLP-1, glucagon-like peptide 1; IL, interleukin; MCP-1, monocyte chemoattractant protein-1; qRT-PCR, quantitative reverse transcription PCR; TAS1R3, taste receptor type 1 member 3; TNFα, tumor necrosis factor-alpha; WD, western diet

As expected, stimulation of NCI-H716 cells with sucrose (glucose + fructose) increased *TAS1R3* mRNA expression; however, *TAS1R3* expression was also increased by palmitate stimulation. Notably, combining palmitate, glucose, and fructose resulted in a larger increase in *TAS1R3* expression than did stimulation with either single molecule (Fig. 2a). The mRNA expression of dimer-forming *TAS1R1* and *TAS1R2* did not differ among groups (see Additional file 2: Fig. S1b, c), consistent with our in vivo findings.

The physiological response of EECs to dietary luminal content mediated by TAS1R3 is the secretion of enteroendocrine hormones, such as GLP-1 [34]. Accordingly, EEC stimulation led to increased *GLP1* mRNA expression and active GLP-1 secretion (Fig. 2b, c), with TAS1R3 more strongly activated by palmitate, glucose, and fructose combined than by either agent alone.

Next, we investigated whether TAS1R3 activation induces the synthesis of pro-inflammatory molecules. Strikingly, the expression levels of interleukin (IL)-1β, IL-6, tumor necrosis factor-alpha (TNF-α), and monocyte chemoattractant protein-1 (MCP-1) were significantly increased across multiple time points and peaking at 12 h upon direct activation of TAS1R3 (Fig. 2d–g). In summary, intestinal TAS1R3 is most potently induced and activated by a combination of luminal fat and sugar, leading to upregulation of pro-inflammatory cytokines and chemokine that can recruit inflammatory cells into the tissues.

To determine whether the enhanced secretion of pro-inflammatory cytokines induced by a combination of glucose, fructose, and palmitate is mediated by TAS1R3 activation, we transfected NCI-H716 cells with siRNA against *TAS1R3*. *TAS1R3* siRNA significantly decreased *TAS1R3* mRNA expression (Fig. 2h, i), resulting in significantly reduced *GLP1* mRNA expression (see Additional file 2: Fig. S1d) and active GLP-1 secretion (Fig. 2j), even with glucose, fructose, and palmitate stimulation. The expression levels of *IL1B*, *IL6*, *IL8*, and *TNF-ɑ* were also significantly reduced by *TAS1R3* knockdown (see Additional file 2: Fig. S1e–h). Notably, the combination of glucose, fructose, and palmitate-induced increases in IL-8 and TNF-ɑ secretion were also inhibited by *TAS1R3* knockdown (Fig. 2k–l).

Consistently, pre-treatment of NCI-H716 cells with lactisole, a TAS1R3 antagonist [35], significantly inhibited the combination of glucose, fructose, and palmitate-induced upregulation of* GLP1* mRNA levels and GLP-1 secretion (see Additional file 2: Fig. S1i and Fig. 2m). Lactisole treatment also prevented the combination of glucose, fructose, and palmitate-induced increase in pro-inflammatory cytokine expression (see Additional file 2: Fig. S1j–m) and secretion (Fig. 2n, o), confirming that a combination of luminal fat and sugar acts through TAS1R3 to induce pro-inflammatory cytokine secretion.

### *Tas1r3*-deficient mice are protected from WD-induced intestinal inflammation

**Fig. 3:**
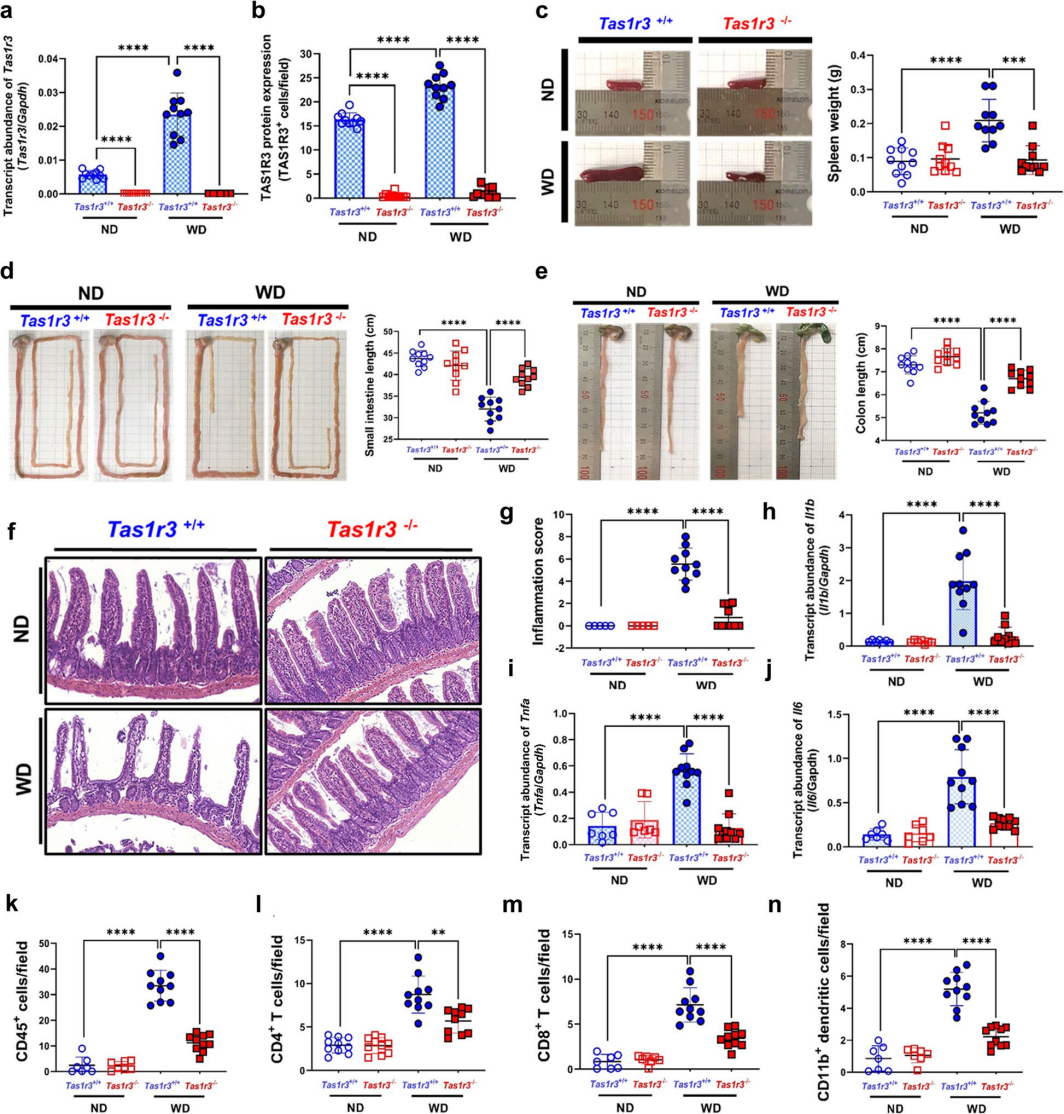
TAS1R3 loss protects against WD-induced intestinal inflammation and reduces inflammatory cell infiltration.** a** qRT-PCR amplification of cDNA from the ileum of *Tas1r3*^+/+^ and *Tas1r3*^−/−^ mice (*n* = 10 mice/group). Ratios of *Tas1r3* to *Gapdh* cycle threshold values are plotted. **b** Immunohistochemical staining of TAS1R3 in terminal ileum tissue. In vivo experiments (*n* = 10 mice/group) were performed in triplicate at least twice. Representative **c** spleens, **d** small intestines, and **e** large intestines from diet-induced *Tas1r3*^+/+^ and *Tas1r3*^−/−^ mice (*n* = 10 mice/group). **f** Hematoxylin and eosin-stained ileal tissue and **g** tissue injury scores in ND- and WD-fed *Tas1r3*^+/+^ and *Tas1r3*^−/−^ mice (*n* = 10 mice/group). qRT-PCR evaluation of **h** *Il1b*, **i** *Il6*, and **j** *Tnfα* in ileal tissue following diet-induced intestinal inflammation in *Tas1r3*^+/+^ and *Tas1r3*^−/−^ mice (*n* = 10 mice/group). *Gapdh* was used as the endogenous control. **k–n** Immunofluorescence and immunohistochemical evaluation of immune cell infiltration in ileal tissue. **k** CD45^+^ leukocytes, **l** CD4^+^ T cells, **m** CD8^+^ T cells, and **n** CD11b^+^ dendritic cells (*n* = 10 mice/group). Data represent means ± standard errors of the mean. **P < 0.01 and ****P < 0.0001 (analysis of variance followed by Bonferroni post-hoc test). ND, normal diet; qRT-PCR, quantitative reverse transcription PCR; TAS1R3, taste receptor type 1 member 3; WD, Western diet

To explore the function of TAS1R3 in intestinal inflammation, we next evaluated WD-induced intestinal inflammation in *Tas1r3*^*−/−*^ mice. TAS1R3 mRNA and protein expression levels were significantly reduced in the ileum of *Tas1r3*^−/−^ mice compared to that in the ileum of wild-type *Tas1r3*^+*/*+^ littermate controls (Fig. 3a, b). We then provided *Tas1r3*^*−/−*^ and *Tas1r3*^+*/*+^ mice with WD or ND ad libitum for 10 weeks and found no significant differences in diet or water intake between them (see Additional file 3: Fig. S2a–c). Moreover, no significant phenotypic differences were detected in spleen weight or small and large intestine length in the ND-fed groups. However, splenomegaly, which indicates increased systemic inflammation, was observed in WD-fed *Tas1r3*^+*/*+^ but not in WD-fed *Tas1r3*^*−/−*^ mice (Fig. 3c). The small and large intestines of WD-fed *Tas1r3*^*−/−*^ mice were significantly longer than those of control mice (Fig. 3d, e).

Next, we examined the histology of intestinal tissues using hematoxylin and eosin staining (Fig. 3f). Although the ND-fed groups were normal, WD-fed *Tas1r3*^*−/−*^ mice displayed fewer epithelial erosions, ulceration, crypt damage, and cellular infiltration in the ileal mucosa than *Tas1r3*^+*/*+^ mice. Histopathological scores also showed attenuation of inflammation in WD-fed *Tas1r3*^*−/−*^ mice relative to that in *Tas1r3*^+*/*+^ mice (Fig. 3g). Intestinal mRNA levels of *Il1b*, *Il6*, and *Tnfa* were significantly lower in WD-fed *Tas1r3*^*−/−*^ mice than in *Tas1r3*^+*/*+^ control mice (Fig. 3h–j).

Pro-inflammatory cytokines can induce an increase in the recruitment of inflammatory cells [36]. In ND-fed *Tas1r3*^*−/−*^ and *Tas1r3*^+*/*+^ mice, inflammatory cells were sparsely present throughout the intestine. However, a marked reduction in their infiltration was observed in the ileum of WD-fed *Tas1r3*^*−/−*^ mice compared to that in WD-fed *Tas1r3*^+*/*+^ mice. The total numbers of CD45^+^ leukocytes, CD4^+^ T cells, CD8^+^ T cells, and CD11b^+^ dendritic cells were significantly lower in the ileum of WD-fed *Tas1r3*^*−/−*^ mice compared to those in *Tas1r3*^+*/*+^ control mice (Fig. 3k–n).

Taken together, the reduction in inflammatory markers in the ileum of WD-fed *Tas1r3*^*−/−*^ mice suggests that TAS1R3 deficiency plays a pivotal role in ameliorating intestinal inflammation.

### *Tas1r3* deficiency modulates intestinal transcriptome

**Fig. 4:**
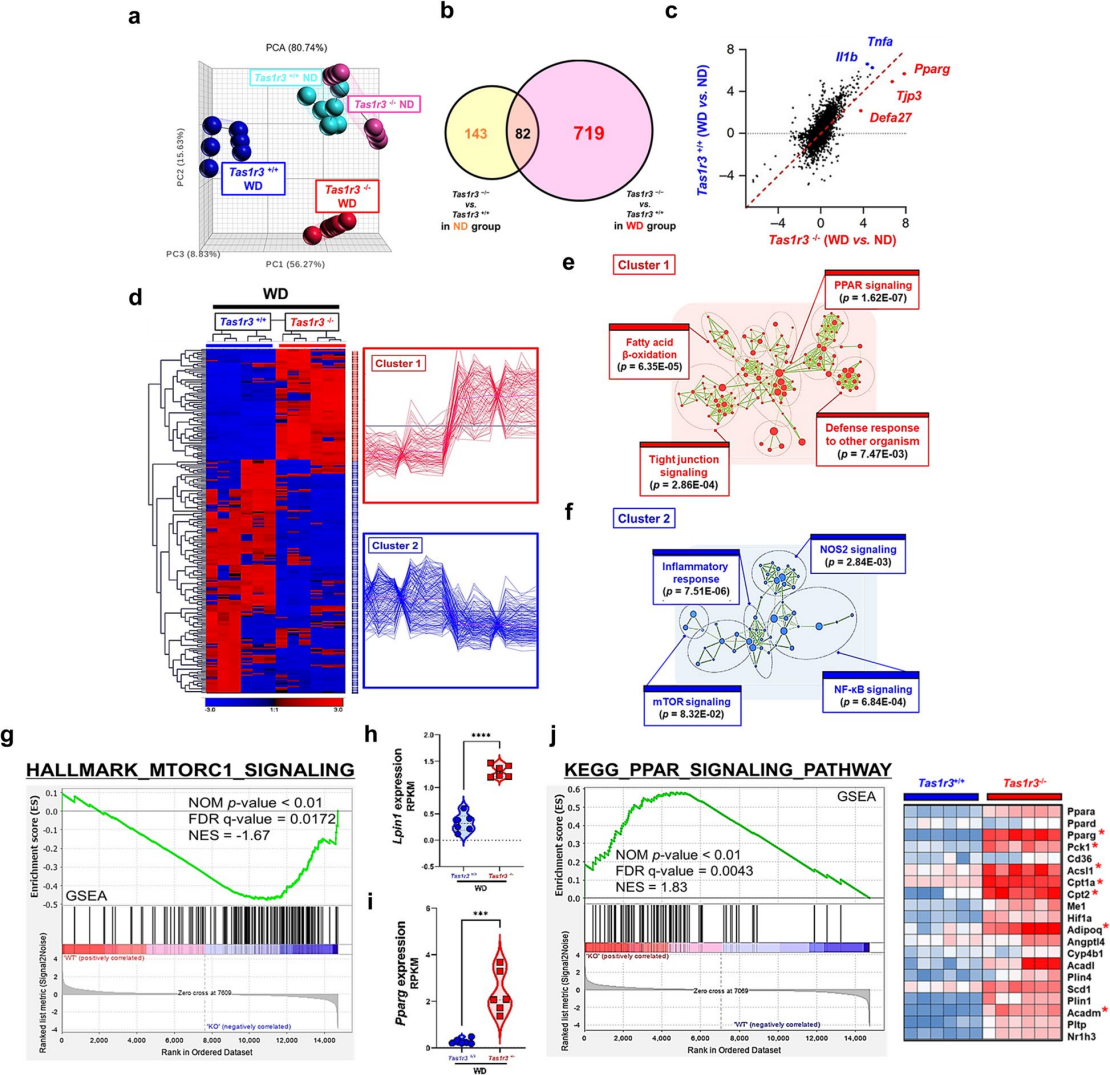
TAS1R3 deficiency modulates the intestinal genome-wide transcriptome.** a** PCA of the ileal transcriptome (*n* = 6 mice/group). Each point represents an individual mouse. **b** DEGs between *Tas1r3*^−/−^ and *Tas1r3*^+/+^ mice in the ND (yellow) and WD (pink) groups. **c** Scatter plots of significantly DEGs between *Tas1r3*^+/+^ and *Tas1r3*^−/−^ mice after adjusting for DEGs with ND in the WD group. **d** Hierarchical clustering and heatmap of up- and downregulated DEGs. Significance [|fold-change|≥ 2, FDR ≤ 0.01] was calculated using Student’s *t*-test. **e** and **f** Seven hundred and nineteen DEGs were subjected to DAVID GO enrichment analysis using Cytoscape with the Enrichment Map plugin. Red or blue nodes and rings represent the top four enriched GO biological pathways for the up- and downregulated genes, respectively. GO pathways with similar functions were clustered together and marked with circles and labels. **g** Gene set enrichment analysis. **h** *Lipin1* and **i** *Pparg* mRNA expression in the small intestine (*n* = 6 mice/group). **j** PPAR signaling pathway gene signature. Genes were ordered by fold-change in descending order from left to right. DEGs enriched in hallmark gene sets are shown in the heatmap. Data represent means ± standard errors of the mean. ***P < 0.001 and ****P < 0.0001 (analysis of variance followed by Bonferroni post-hoc test). DEG, differentially expressed gene; FDR, false discovery rate; GO, Gene Ontology; ND, normal diet; PCA, principal component analysis; TAS1R3, taste receptor type 1 member 3; WD, Western diet

To understand how TAS1R3 deficiency alleviates intestinal inflammation, we investigated transcriptional changes in the ileum of ND- and WD-fed *Tas1r3*^*−/−*^ and *Tas1r3*^+*/*+^ mice using RNA-seq. PCA confirmed that the gene expression profiles of ND-fed *Tas1r3*^*−/−*^ and *Tas1r3*^+*/*+^ mice were similar, whereas those of WD-fed *Tas1r3*^−/−^ mice were easily distinguished from those of WD-fed *Tas1r3*^+*/*+^ control mice (Fig. 4a). Although only 143 DEGs were identified between ND-fed *Tas1r3*^*−/−*^ and *Tas1r3*^+*/*+^ mice [|fold-change|≥ 2, FDR ≤ 0.01], 719 DEGs were identified between the WD-fed groups, suggesting that TAS1R3 has a stronger effect in the presence of WD-induced inflammatory progression (Fig. 4b).

The transcription of genes encoding inflammatory cytokines, such as *Tnfa* and *Il1b*, was increased in WD-fed *Tas1r3*^+*/*+^ mice, whereas the expression of *Pparg*, *Tjp3*, and *Defa27* was increased in WD-fed *Tas1r3*^*−/−*^ mice (Fig. 4c). Hierarchical clustering revealed two distinct gene clusters (Fig. 4d). Cluster 1, which comprised prominent upregulated genes in WD-fed *Tas1r3*^*−/−*^ mice, was further subdivided to assess biological processes. Functional classification and Fisher’s exact test identified PPAR signaling (*P* = 1.62E-07), fatty acid β-oxidation (*P* = 6.35E-05), tight-junction signaling (*P* = 2.86E-04), and defense response to other organisms (*P* = 7.47E-03) as the most significant biological processes (Fig. 4e). Meanwhile, the most enriched biological processes in Cluster 2, comprising downregulated genes in *Tas1r3*^*−/−*^ mice, were inflammatory response (*P* = 7.51E-06), NF-κB signaling (*P* = 6.84E-04), NOS2 signaling (*P* = 2.84E-03), and mTOR signaling (*P* = 8.32E-02) (Fig. 4f). We confirmed that the absence of TAS1R3 significantly reduced inflammatory signaling. Moreover, GSEA plots revealed that mTOR signaling was significantly suppressed (Fig. 4g).

Among the genes encoding downstream mTOR components, *Lpin1* and *Pparg* were expressed at high levels in *Tas1r3*-deficient mice (Fig. 4h, i). GSEA confirmed that the expression of genes associated with the PPAR signaling pathway (*Pparg*, *Pck1*, *Acsl1*, *Cpt1a*, *Cpt2*, *Adopoq*, and *Acadm*) was significantly enriched in the set of DEGs between *Tas1r3*^*−/−*^ and *Tas1r3*^+*/*+^ mice fed the WD (FDR q < 0.01; Fig. 4j), suggesting that TAS1R3 functions as an important regulator of PPAR-γ signaling in the intestinal tract, due to its suppression of mTOR.

### TAS1R3 controls pro-inflammatory cytokine secretion by regulating the mTOR–PPARγ axis in EECs

**Fig. 5:**
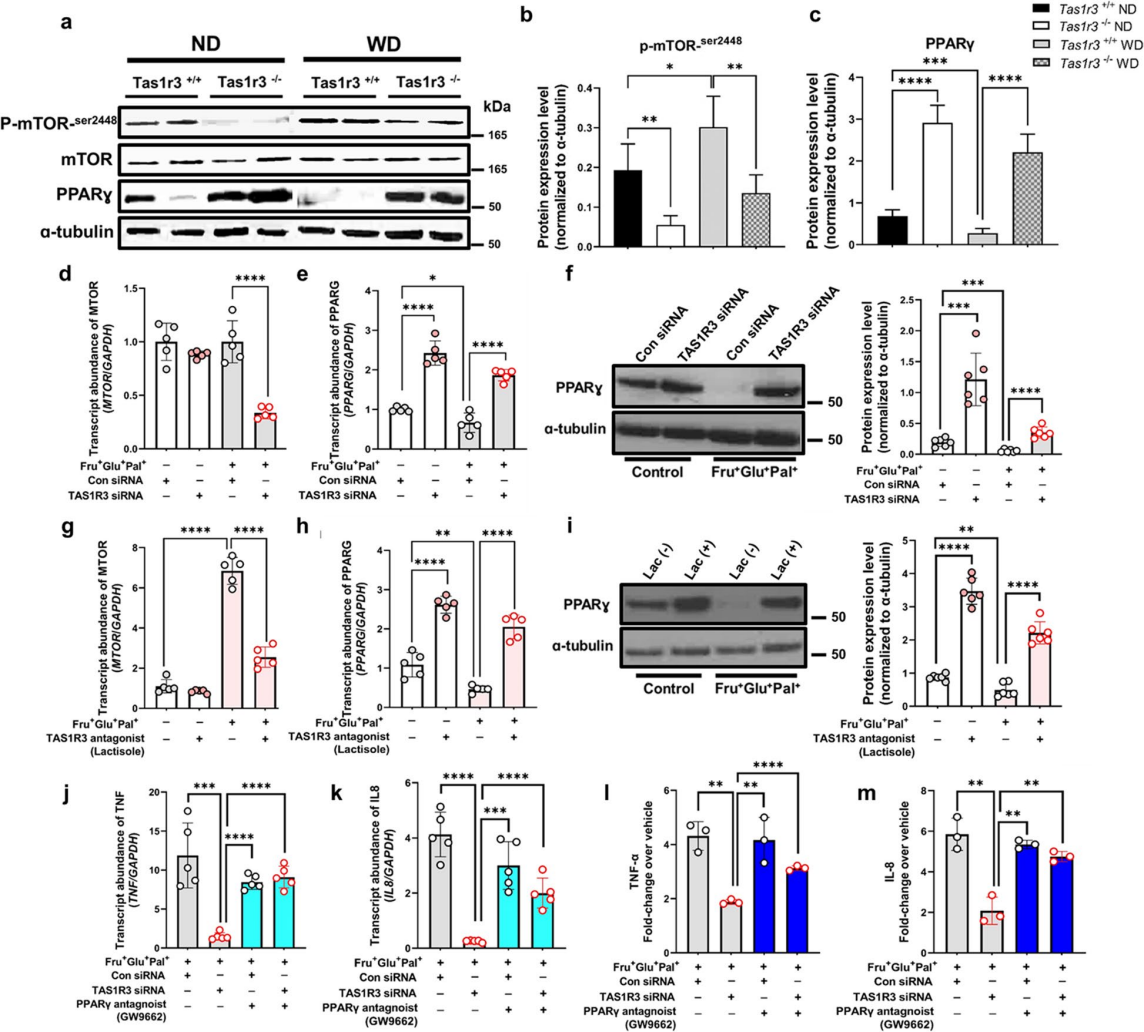
TAS1R3 controls pro-inflammatory cytokine production and secretion by regulating the mTOR–PPARγ axis. Western blotting of (**a**) phospho-mTOR, mTOR, and PPARγ expression in ND- and WD-fed *Tas1r3*^−/−^ and *Tas1r3*^+/+^ mice and (**b**) and (**c**) densitometric data (*n* = 6 mice/group). Representative images of at least three different blots and α-tubulin. **d–f** NCI-H716 cells were transfected with *TAS1R3* (10 nM) or scrambled control (10 nM) siRNA for 48 h and stimulated with or without fructose (10 mM), glucose (10 mM), and palmitate (10 μM) for 12 h. qRT-PCR analysis of (**d**) *MTOR* and (**e**) *PPARG* mRNA expression relative to *GAPDH* (*n* = 5/group). **g–i** NCI-H716 cells were pretreated with the TAS1R3 antagonist, lactisole (2.5 mM), for 30 min and stimulated with fructose (10 mM), glucose (10 mM), and palmitate (10 μM) for 12 h. qRT-PCR analysis of (**g**) *MTOR* and (**h**) *PPARG* mRNA expression relative to *GAPDH* (*n* = 5/group). **f** and **i** PPARγ protein abundance was assessed by western blotting (*n* = 6/group). Alpha-tubulin was used as the loading control. **j–m** NCI-H716 cells were transfected with *TAS1R3* (10 nM) or scrambled control (10 nM) siRNA for 48 h and stimulated with the PPARγ antagonist, GW9662 (10 μM), in the presence of fructose (10 mM), glucose (10 mM) and palmitate (10 μM) for 12 h. qRT-PCR analysis of (**j**) *TNFA* and (**k**) *IL8* mRNA expression relative to *GAPDH* (*n* = 5/group) and enzyme-linked immunosorbent assay of (**l**) TNF-α and (**m**) IL-8 protein abundance (*n* = 3/group). Data represent means ± standard errors of the mean of three to five independent experiments. *P < 0.05, **P < 0.01, ***P < 0.001, and ****P < 0.0001 (one-way analysis of variance followed by Bonferroni post-hoc test). ND, normal diet; qRT-PCR, quantitative reverse transcription PCR; TAS1R3, taste receptor type 1 member 3; WD, Western diet

To validate whether changes in the transcript levels of mTOR and PPARγ regulated by TAS1R3 deficiency were also present at the protein level, western blotting was performed. Consistently, intestinal PPARG protein was expressed at higher levels in *Tas1r3*^−/−^ mice than in *Tas1r3*^+/+^ control mice. Moreover, PPARG expression was decreased by WD intake in *Tas1r3*^+/+^ mice, whereas it remained significantly increased in *Tas1r3*^−/−^ mice (Fig. 5a–c). We found that reduced expression of phosphorylated mTOR (a modulator of PPARγ) occurred in conjunction with elevated PPARγ levels in *Tas1r3*^−/−^ mice (Fig. 5a, b), indicating a potential crosstalk between mTOR and PPARγ signaling.

Next, we sought to determine whether TAS1R3 directly regulates the mTOR–PPARγ axis in vitro EECs (Fig. 5d–m). *MTOR* expression was significantly increased under the Fru^+^Glu^+^Pal^+^ condition and significantly decreased when *TAS1R3* expression was suppressed by siRNA or lactisole treatment (Fig. 5d, g). Additionally, *PPARG* mRNA and protein expression continuously increased following *TAS1R3* suppression, regardless of the nutritional condition (Fig. 5e–i).

PPARγ is expressed at high levels in the intestinal tract, and its activation inhibits the production of numerous inflammatory cytokines through its action on kinases and transcription factors, including NF-κB, c-Jun, and c-Fos [37, 38]. To investigate whether TAS1R3 induces the production and secretion of pro-inflammatory cytokines via PPARγ regulation, the increase in PPARγ signaling caused by *TAS1R3* suppression was blocked by the PPARγ antagonist, GW9662. Interestingly, the production and secretion of TNF and IL-8, which were reduced following TAS1R3 suppression, were rescued by GW9662 (Fig. 5j–m).

Taken together, these results suggest that TAS1R3 acts as an intrinsic regulator of PPARγ within EECs, thereby regulating the expression and secretion of pro-inflammatory cytokines.

### TAS1R3 deficiency alters the composition of the gut microbiome

The nuclear transcription factor, PPARγ, also exerts an anti-inflammatory effect by regulating the expression of genes encoding tight-junction proteins (TJPs) and antimicrobial peptides (AMPs) [39–41].

**Fig. 6:**
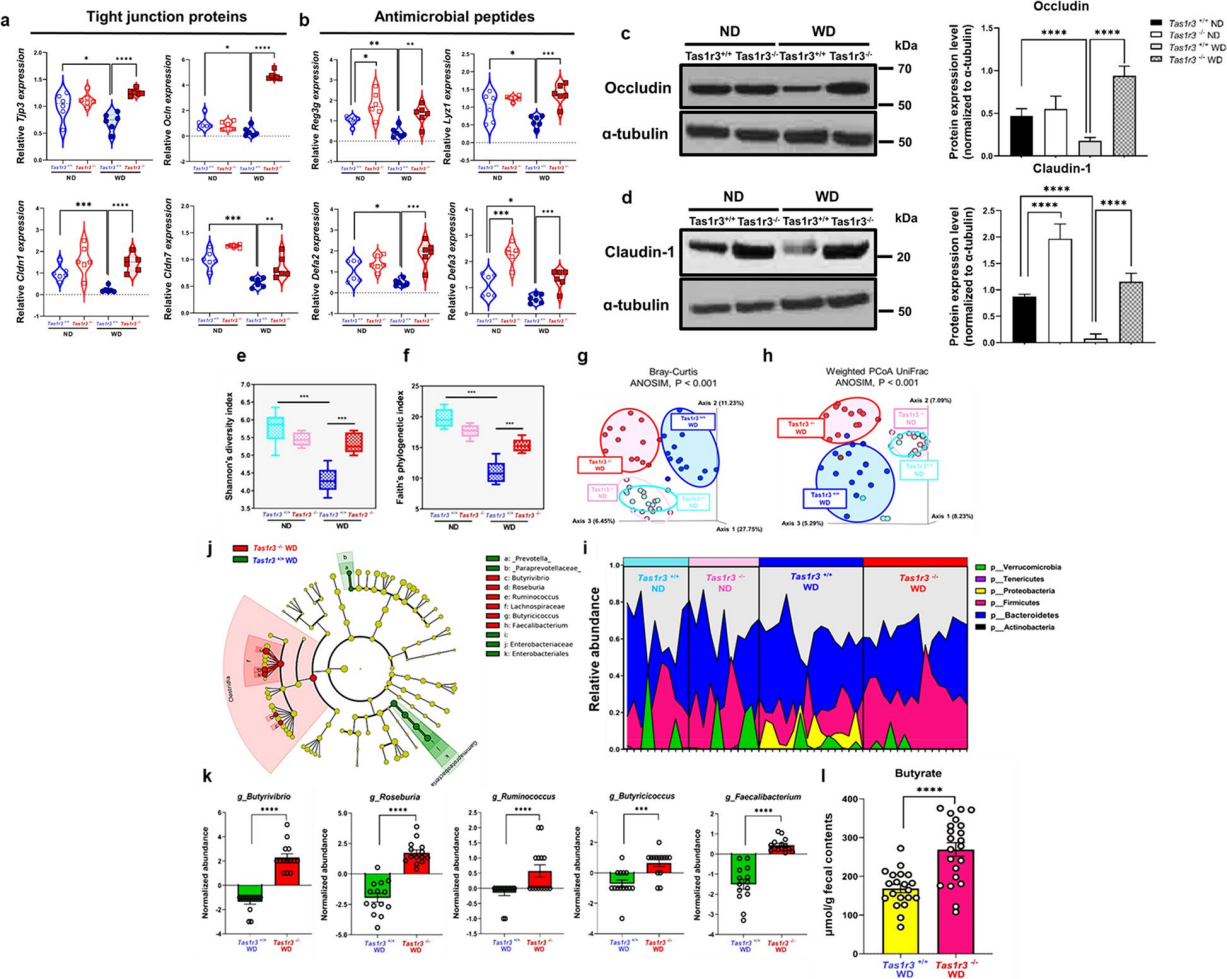
TAS1R3 deficiency alters the gut microbiota composition and increases the abundance of butyrate-producing bacteria.** a** and **b** mRNA expression of PPARγ-target genes (TJPs: *Tjp3*, *Ocln*, *Cldn1*, and *Cldn7*; AMPs: *Reg3g*, *Lyz1*, *Defa2*, and *Defa3*) in the small intestine (*n* = 6 mice/group). Western blotting of (**c**) Occludin and (**d**) Claudin-1 expression in ND- and WD-fed *Tas1r3*^−/−^ and *Tas1r3*^+/+^ mice and densitometric data (*n* = 6 mice/group). **e–h** Effects of TAS1R3 deficiency on the α- and β-diversity of the gut microbiota (*n* = 10–14 mice/group). **e** and **f** Changes in intestinal bacterial community diversity, determined by Shannon’s and Pielou’s indices. **g** and **h** Three-dimensional PCA based on Bray − Curtis and weighted UniFrac distances between all samples. Each circle represents an individual mouse. Significance was tested using PERMANOVA. **i** Relative abundance of bacterial phyla. Each color represents the relative abundance of a specific phylum in each sample (*n* = 10–14 mice/group). Only phyla with a mean relative abundance of > 0.1% are shown. **j** Comparison of bacterial community composition between WD-fed *Tas1r3*^−/−^ and *Tas1r3*^+/+^ mice. Taxonomic cladogram generated from linear discriminant analysis effect size analysis of gut microbial 16S rRNA sequencing data. Taxa meeting a linear discriminant analysis threshold of > 2 and *P* < 0.01 are shown. **k** Relative abundance of genera differentially detected between the groups (*n* = 14 mice/group). **l** Fecal butyrate levels (μM/g wet weight) in WD-fed *Tas1r3*^−/−^ and *Tas1r3*^+/+^ mice, assessed by gas chromatography-mass spectrometry (*n* = 20 mice/group). Data represent means ± standard errors of the mean. *P < 0.05, **P < 0.01, ***P < 0.001, and ****P < 0.0001 (analysis of variance followed by Bonferroni post-hoc test or unpaired Student’s *t*-test). AMP, antimicrobial peptide; PCA, principal coordinate analysis; TAS1R3, taste receptor type 1 member 3; TJP, tight-junction protein; WD, western diet

To confirm whether TAS1R3 deficiency alters PPARγ transcriptional activity, we screened the expression of PPARγ-regulated genes in vivo. Elevated expression of PPARγ by TAS1R3 deficiency resulted in a significant increase in the expression of TJP genes, including *Tjp3*, *Ocln*, *Cldn1*, and *Cldn7*, as well as AMP genes, including *Reg3g*, *Lyz1*, *Defa2*, and *Defa3*, all of which are regulated by PPARγ (Fig. 6a, b). Consistently, the intestinal tight junction proteins Occludin and Claudin-1 were expressed at higher levels in *Tas1r3*^−/−^ mice than in *Tas1r3*^+/+^ control mice (Fig. 6c, d). These gut barrier proteins were decreased by WD intake in *Tas1r3*^+/+^ mice, whereas they remained significantly increased in *Tas1r3*^−/−^ mice.

The upregulation of both TJPs and AMPs in *Tas1r3*^*−/−*^ mice prompted us to investigate how TAS1R3 deficiency affects the gut microbial community structure. To this end, we performed high-throughput sequencing analysis of 16S rRNA in fecal bacterial DNA isolated from *Tas1r3*^*−/−*^ mice and wild-type littermate controls fed the ND or WD. The α-diversity analysis showed that WD significantly reduced species richness in *Tas1r3*^+*/*+^ mice. However, no changes in α-diversity were observed in *Tas1r3*^*−/−*^ mice between WD and ND (Fig. 6e, f). The β-diversity of the microbial population was also significantly altered in the WD-fed groups, reflected by distinct clustering patterns in the principal coordinate analysis (PCoA) plots (Fig. 6g, h), whereas ND-fed *Tas1r3*^*−/−*^ and *Tas1r3*^+*/*+^ mice exhibited similar profiles, suggesting that TAS1R3 significantly altered the gut microbiota composition in the context of WD intake.

At the phylum level, the relative abundance of Proteobacteria, a characteristic feature of IBD in mice and humans [42], was significantly high in WD-fed *Tas1r3*^+*/*+^ mice compared to that in ND-fed *Tas1r3*^+/+^ mice (Fig. 6i). Linear discriminant analysis effect size analysis further revealed that abundances of major discriminators, such as Paraprevotellaceae, *Prevotella*, Enterobacteriaceae, and Enterobacteriales, which are well-known pathobionts implicated in irritable bowel syndrome and IBD [43], were significantly higher in WD-fed *Tas1r3*^+*/*+^ mice (Fig. 6j). These results indicate that long-term WD intake enhances the expansion of IBD-related pathobionts.

In contrast, the relative abundance of Firmicutes was markedly high in WD-fed *Tas1r3*^*−/−*^ mice compared to that in WD-fed *Tas1r3*^+/+^ mice (Fig. 6i). This enrichment was further confirmed for Firmicutes subclades using a phylogenetic cladogram (Fig. 6j). TAS1R3 deficiency significantly enhanced the relative abundance of Lachnospiraceae, *Butyrivibrio*, *Roseburia*, *Ruminococcus*, *Butyricicoccus*, and *Faecalibacterium* (all the Clostridia; Fig. 6k); these bacteria all contributed to the increased abundance of Firmicutes in WD-fed *Tas1r3*^*−/−*^ mice. Importantly, the latter five genera are obligate anaerobic butyrate-producers [44]. Butyrate is the major end-product of bacterial fermentation and regulates gut inflammation by maintaining intestinal mucosal integrity [45]. Given that TAS1R3 deficiency significantly increased the proportion of butyrate-producing Clostridia compared to that in WD-fed *Tas1r3*^+/+^mice, we performed gas chromatography–mass spectrometry to quantify fecal butyrate. Butyrate content was significantly higher in WD-fed *Tas1r3*^*−/−*^ than in *Tas1r3*^+*/*+^ mice (Fig. 6l). These results indicate that TAS1R3 deficiency alters the composition of the gut microbiome by increasing the abundance of butyrate-producing bacteria, which may improve intestinal mucosal integrity and contribute to the maintenance of intestinal homeostasis.

### Host *Pparg* expression positively correlates with the abundance of obligate anaerobic butyrate-producing bacteria

**Fig. 7:**
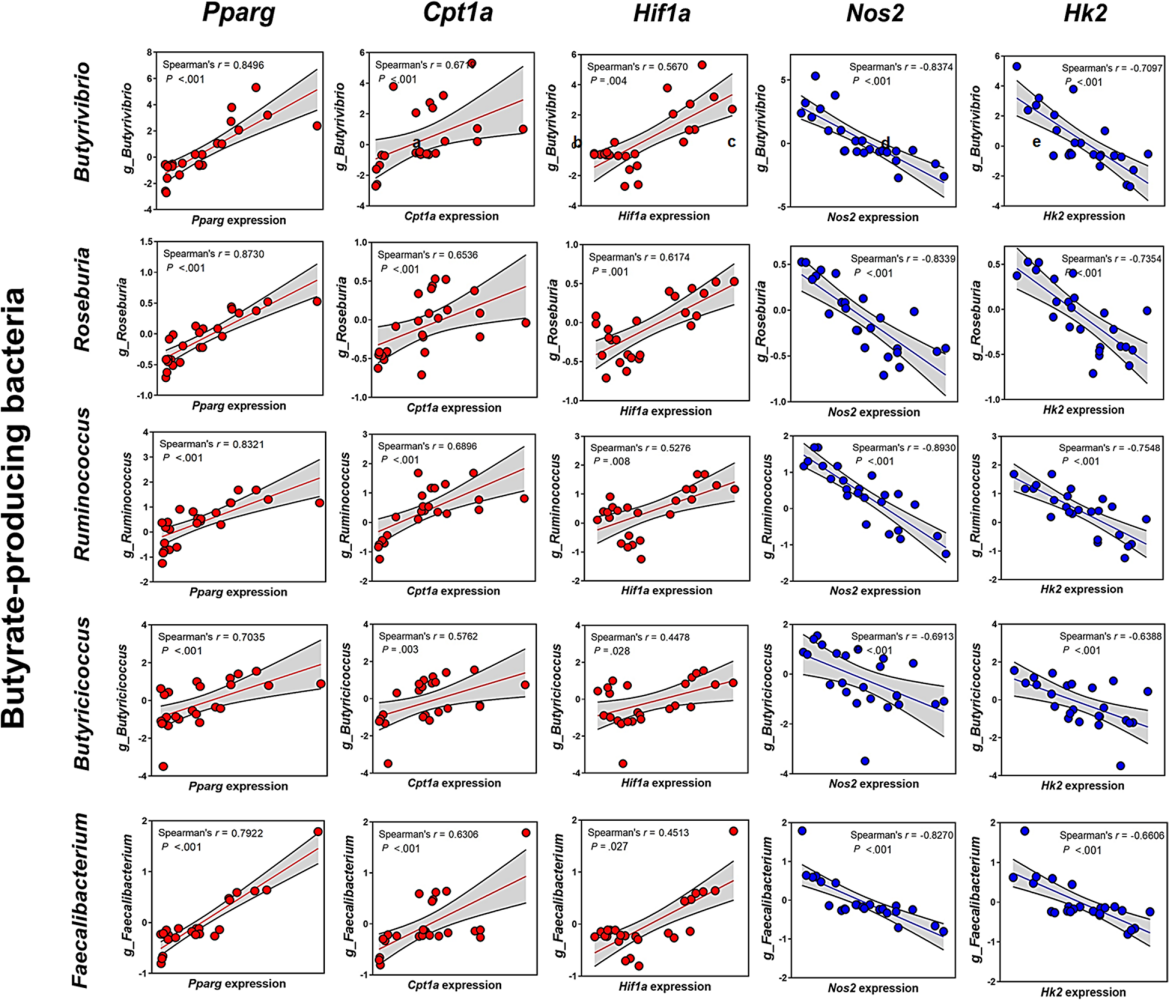
Crosstalk between host intestinal PPARγ signaling-related genes and obligate anaerobic butyrate-producing gut microbes. Interactions between host intestinal (**a**) *Pparg*, (**b**) *Cpt1a*, (**c**) *Hif1a*, (**d**) *Nos2*, and (**e**) *Hk2* and obligate anaerobic butyrate-producing bacteria (*Butyrivibrio*, *Roseburia*, *Ruminococcus*, *Butyricicoccus*, and *Faecalibacterium*) (*n* = 6 mice/group). Red and blue points indicate positive and negative correlations, respectively; all correlations shown are significant

To determine whether the increased abundance of butyrate-producing bacteria in WD-fed *Tas1r3*^*−/−*^ mice was associated with the increase in host PPARγ expression, we performed host-microbial correlation analysis. Host *Pparg* expression was positively correlated with the abundance of dominant obligate anaerobic butyrate-producing taxa that had exhibited altered abundance in WD-fed *Tas1r3*^*−/−*^mice (Fig. 7a).

As intestinal PPARγ signaling plays a role in limiting oxygen bioavailability in the gut lumen [46, 47], in addition to serving as a transcription factor, we conducted further Spearman correlation analyses. The expression levels of *Cpt1a* (a major β-oxidation marker) and *Hif1a* (a marker of hypoxia) were significantly and positively correlated with the dominant obligate anaerobic butyrate-producing bacteria, whereas the expression levels of *Nos2* (an indicator of aerobic glycolysis) and *Hk2* (a key mediator of aerobic glycolysis) were significantly and negatively correlated with the abundance of the dominant obligate anaerobic butyrate-producing bacteria (Fig. 7b–e).

### TAS1R3 promotes intestinal inflammation in patients with IBD and mice with colitis

**Fig. 8:**
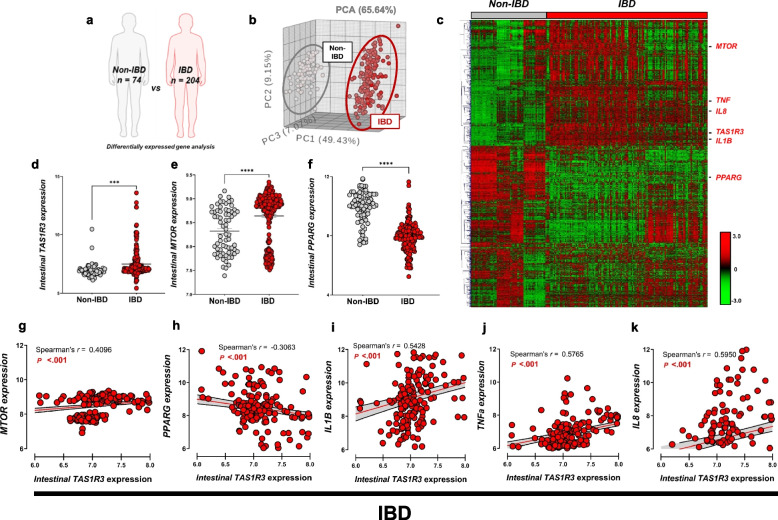
TAS1R3 promotes intestinal inflammation in patients with IBD. **a** Transcriptome meta-analysis. Only datasets preprocessed and normalized in one step were used (GSE160804/GSE126124/GSE95095/GSE75214/GSE53306). **b** PCA of intestinal biopsy transcriptomes from patients with and without IBD. Grey and red points represent individual IBD (*n* = 204) and non-IBD (*n* = 74) samples, respectively. **c** Hierarchical clustering and heatmap of up- and downregulated genes. A total of 1,697 significantly DEGs were identified (unpaired Student’s *t*-test; FDR *P* < 0.001). **d** *TAS1R3*, **e** *MTOR*, and **f** *PPARG* mRNA expression in intestinal biopsies from patients with (*n* = 204) and without IBD (*n* = 74). Spearman’s correlation analysis of associations between intestinal *TAS1R3* mRNA expression and that of **g** *MTOR* and **h** *PPARG*, as well as pro-inflammatory cytokines: **i** *IL1B*, **j** *TNFa*, and **k** *IL8*. All correlations shown are significant. Data are expressed as means ± standard errors of the mean. *P < 0.05, **P < 0.01, ***P < 0.001, and ****P < 0.0001 (unpaired Student’s *t*-test). DEF, differentially expressed gene; FDR, false discovery rate; IBD, inflammatory bowel disease; PCA, principal coordinate analysis

To investigate the clinical relevance of our findings, we compared the gene expression profiles of intestinal biopsies between patients with (*n* = 204) and without (*n* = 74) IBD from the publicly available Gene Expression Omnibus database (Fig. 8a). As shown in the PCA plot (Fig. 8b), the intestinal gene expression profiles of patients with and without IBD were clearly distinguishable. Among the 1,697 upregulated DEGs (FDR *P* < 0.001) in patients with IBD, *TAS1R3* expression was increased in the intestinal tract (Fig. 8c, d), as was the expression of *TNFA*, *IL1B*, and *IL8* (Fig. 8c). Moreover, the expression of *MTOR* was upregulated (Fig. 8e), whereas that of *PPARG* was downregulated in the intestines of patients with IBD (Fig. 8f). Intestinal *TAS1R3* expression in patients with IBD was significantly and positively correlated with *MTOR* expression, and negatively correlated with *PPARG* expression (Fig. 8g, h). Furthermore, higher intestinal *TAS1R3* expression correlated with a higher abundance of IL-1β, IL-8, and TNF-α (Fig. 8i–k).

We also assessed the effects of *Tas1r3* deficiency on the development of intestinal inflammation in a DSS-induced colitis mouse model. *Tas1r3*^*−/−*^ mice and *Tas1r3*^+*/*+^ control mice were administered 2% DSS in their drinking water for 7 days. The daily water intake did not significantly differ between groups. However, *Tas1r3*^+*/*+^ mice exhibited accelerated and severe weight loss compared to *Tas1r3*^*−/−*^ mice, during DSS administration (see Additional file 4: Fig. S3a). The disease activity indices of *Tas1r3*^+*/*+^ mice, including severe diarrhea and intestinal bleeding, were significantly higher (see Additional file 4: Fig. S3b), and the colon length substantially shorter, than those of *Tas1r3*^*−/−*^ mice (see Additional file 4: Fig. S3c). DSS caused severe small and large intestinal tissue damage and significantly higher inflammation scores in *Tas1r3*^+*/*+^ mice than in *Tas1r3*^*−/−*^ mice (see Additional file 4: Fig. S3d, e). After 7 days of DSS treatment, a marked increase in immune cell infiltration was observed, with a significant increase in the total number of intestinal leukocytes in the small and large intestines of *Tas1r3*^+*/*+^ mice compared with *Tas1r3*^*−/−*^ mice (see Additional file 4: Fig. S3f, g).

Taken together, these findings indicate that the severity of DSS-induced colitis was significantly decreased in *Tas1r3*^*−/−*^ mice, suggesting that TAS1R3 deficiency alleviates intestinal inflammation.

## Discussion

In this study, we extensively analyzed the role of taste receptor TAS1R3 in bowel tissue inflamed by prolonged ingestion of WD. Our results showed that the nutrient-sensing intestinal receptor, TAS1R3, is a key modulator of host-gut microbial interactions, ultimately regulating intestinal inflammation (see Additional file 5: Fig. S4).

In contrast to chemosensory tuft cells, which sense parasite- or metabolite-derived ligands and modulate the immune system in response [19, 20, 47, 48], EECs are key chemosensory cells that directly sense nutrient ligands and exhibit spatial heterogeneity with tuft cells. Herein, we found that EECs express TAS1R3 and directly recognize endogenous nutrient ligands to regulate the secretion of pro-inflammatory molecules, suggesting that they serve as key agents in the pathogenesis of IBD. Indeed, one of our primary findings is that nutrients, rather than microorganisms, directly prompt EECs to secrete cytokines via nutrient-sensing TRs. Moreover, although TAS1R3 ligands in the oral cavity were previously considered to only be sweet molecules [33], herein we provide evidence that intestinal TAS1R3 responds to dietary fat as well. Furthermore, we demonstrate that robust pro-inflammatory cytokines secreted in EECs via TAS1R3 activation by nutrient binding can ultimately attract multiple inflammatory cells, thereby worsening intestinal inflammation. Consistent with this, pro-inflammatory cytokines, such as TNF, are key targets for IBD therapy [49]. New agents that target cytokines, or their signaling cascades, are currently being tested in clinical trials, suggesting that cytokine blockade is a promising approach for IBD therapy. Thus, our findings may have important applications for IBD management.

WD intake promotes the expansion of pro-inflammatory pathogenic gut bacteria, ultimately leading to gut dysbiosis [50]. Here, we found that at the phylum level, the relative abundance of Proteobacteria, which is expanded in mouse and human IBD [42], was significantly high in WD-fed wild-type (*Tas1r3*^+*/*+^) mice compared to that in ND-fed *Tas1r3*^+/+^ mice. Moreover, the abundance of major discriminators (well-known pathobionts associated with irritable bowel syndrome and IBD) [51, 52], significantly increased. Hence, long-term WD intake enhances the expansion of IBD-associated pathobionts, consistent with our model of WD-induced intestinal inflammation. In contrast, WD-induced gut dysbiosis did not occur in *Tas1r3*-deficient mice with increased PPARγ as a downstream target. Elevated PPARγ expression induced by TAS1R3 deficiency may protect against gut dysbiosis through two main mechanisms: (1) driving β-oxidation, leading to the expansion of obligate anaerobic butyrate-producing bacteria, by maintaining a hypoxic state, with obligate anaerobic butyrate-producing bacteria suppressing the dysbiotic expansion of facultative anaerobic pathogenic bacteria by competing for oxygen; and (2) directly regulating the mTOR–PPARγ axis in the intestine, by an intrinsic mechanism, reflected by the markedly increased abundance of intestinal PPARγ in ND-fed *Tas1r3*-deficient mice, which may protect against dysbiosis by strengthening gut barrier function. Overall, our results indicate that gut TAS1R3 is an intrinsic regulator of intestinal inflammation and constitutes a central mechanism for controlling intestinal crosstalk with the gut microbiota.

PPARγ regulates intestinal inflammation [53–55]. Another novel finding of this study is the identification of PPARγ as a downstream target regulated by TAS1R3 deficiency. Genetic ablation of PPARγ results in increased susceptibility to experimental colitis in mice, supporting its anti-inflammatory properties [54]. In contrast, PPARγ signaling agonists, such as rosiglitazone, attenuate the severity of inflammatory lesions in both experimental and spontaneous models of colitis [55]. Although the anti-inflammatory mechanism of PPARγ remains unclear, findings from previous studies suggest the following: (i) PPARγ activation inhibits the production of numerous inflammatory cytokines, through its action on kinases and transcription factors, such as NF-κB, c-Jun, and c-Fos [37, 38, 56]; (ii) PPARγ agonists enhance barrier function by upregulating tight-junction molecules in gastrointestinal epithelial cells and maintaining intestinal mucosal integrity [39, 57, 58]; and (iii) PPARγ may exert key antimicrobial effects, by activating AMP production, including intestinal α- and β-defensins [40, 41]. Consistent with these mechanisms, we found that *Tas1r3*-deficient mice exhibited increased expression of intestinal PPARγ, leading to significantly increased expression of TJPs, and several AMPs, along with decreased expression of pro-inflammatory cytokines (TNF-α, IL-1β, IL-6, and IL-8). Furthermore, PPARγ inhibition via GW9662 in TAS1R3 knockdown cells rescued the secretion of pro-inflammatory cytokines induced by the blockade of TAS1R3. Thus, *TAS1R3* deficiency plays an important role in regulating the inflammatory response through PPARγ. In turn, PPARγ-dependent processes, through which TAS1R3 mediates its effects, may alleviate intestinal inflammation.

The regulation of intestinal PPARγ expression and host intestinal-microbial interactions are also closely linked [59]. PPARγ drives the energy metabolism of intestinal epithelial cells towards β-oxidation and enhances oxygen consumption while suppressing iNOS synthesis. Therefore, PPARγ signaling leads to decreased oxygen bioavailability in the gut lumen [46]. In a hypoxic environment, the gut microbiota is dominated by obligate anaerobic bacteria, further limiting the generation of host-derived nitrogen and oxygen, preventing dysbiotic expansion of potentially pathogenic facultative anaerobic bacteria by competing for oxygen, resulting in the maintenance of healthy gut homeostasis [47]. Conversely, inhibition of epithelial PPARγ signaling leads to metabolic reorientation of enterocytes towards aerobic glycolysis rather than β-oxidation, increasing epithelial oxygenation and elevating oxygen bioavailability to promote the expansion of facultative anaerobic bacteria [47]. Accordingly, we confirmed that prolonged exposure to a WD resulted in the expansion of facultative pathogenic anaerobic bacteria, such as *Enterobacteriaceae* and *Prevotellaceae*, in low PPARγ-expressing *Tas1r3*^+*/*+^ mice. However, *Tas1r3*-deficient mice, with high PPARγ expression, harbored predominantly obligate anaerobic bacteria, even with the consumption of WD.

The drive towards β-oxidation, reinforced through PPARγ signaling under TAS1R3 deficiency, was positively correlated with the abundance of obligate anaerobic bacteria, such as butyrate-producing bacteria. Thus, epithelial hypoxia occurring consequent to host PPARγ expression maintains anaerobiosis in the gut to support the dominance of butyrate-producing bacteria. Moreover, luminal butyrate functions as a major driver of the PPARγ-dependent transcriptional response in intestinal epithelial cells [46], constituting a positive feedback loop that substantially amplifies the protective effects against intestinal inflammation [60, 61]. Our findings show that TAS1R3 acts as a coordinating hub for host enteric defense through crosstalk between bacteria and intestinal epithelial cells.

In humans, reduced PPARγ expression in intestinal epithelial cells is a hallmark of IBD [62, 63]. However, the mechanism underlying this reduction remains poorly understood. Here, we report the co-occurrence of increased TAS1R3 and reduced PPARG expression in intestinal biopsies of patients with IBD. These clinical data are consistent with our animal model findings. In addition to the WD-induced inflammation model, *Tas1r3* deficiency suppressed intestinal inflammation in a DSS-induced colitis model. This is a widely used model exhibiting several characteristics of human IBD [23]. Therefore, our findings provide strong evidence that TAS1R3 contributes to IBD in humans and mice.

We also found that *Tas1r3*-deficient mice had significantly lower mTOR activity in the intestinal tract than wild-type mice, following the consumption of WD. Notably, low nutrient conditions suppress mTORC1, leading to increased rates of catabolic processes [64, 65]. The absence of the nutrient-sensing TR, TAS1R3, may induce a nutrient-starvation-causing scenario. Thus, despite nutrient replete conditions (i.e., prolonged WD), *TAS1R3*-knockout enhanced catabolism, because of the perceived starvation. This assumption is supported by the upregulation of mRNAs encoding the catabolic markers, Lipin1 and PPARγ, within the repressed mTOR signaling pathway. That is, inhibition of mTOR induces Lipin1 activation, thereby activating PPARγ, which inhibits lipogenesis and drives intracellular metabolism towards β-oxidation [66, 67]. This was consistently observed in *Tas1r3*-deficient mice; thus, TAS1R3 likely acts as a nutrient sensor that conveys early signals regarding nutrient availability to the energy-sensing component of mTOR.

A focus of this study was to assess the role of environmental factors in etiology of IBD. Our animal model findings confirmed that the consumption of a WD is a major trigger of intestinal inflammation, with gut-expressed TAS1R3 representing a key target of WD-induced inflammation. However, although intestinal TAS1R3 expression was markedly increased in patients with IBD, we did not determine whether WD intake directly increased TAS1R3 expression, due to the absence of dietary records. Further studies are needed to analyze habitual dietary patterns to better assess the associations between WD components and TAS1R3 overactivation in patients with IBD. Particularly, the expression of TAS1R3 as a function of WD intake should be monitored in patients during the pre-IBD to IBD transition. Furthermore, it might be needed for future work to look at how microbial changes in *Tas1r3*-deficient mice can affect colitis susceptibility by transferring altered microbial contents to germ-free or antibiotic mice.

## Conclusions

Intestinal *Tas1r3* deficiency modulates the response to luminal dietary stimuli via the transcription factor PPARγ, which regulates intestinal mucosal defense. These host defense mechanisms interact closely with butyrate-producing gut microbiota. Finally, the mRNA expression of TAS1R3 and PPARγ is aberrantly regulated in patients with IBD. A key finding of our study is that nutrient molecules, rather than microorganisms, directly prompt EECs to secrete cytokines via nutrient-sensing TRs. Moreover, TAS1R3 may be a key regulator of diet-induced interactions between the host and gut microbiome. Considering that membrane receptors are readily accessible to drugs, TAS1R3 may be a valuable therapeutic target for IBD. These findings provide a foundation for determining the undesirable effects of WD on intestinal inflammation and new insights into the development of strategies aimed at preventing and managing IBD.

## Supplementary Information

**Additional file 1: Table S1.** siRNA target sequences. **Table S2.** RT-PCR primers. **Table S3.** List of transcriptome datasets retrieved from publicly available Gene Expression Omnibus. **Table S4.** Descriptive statistics.

**Additional file 2: Figure S1.** The effect of inhibition of TAS1R3 by siRNA transfection and antagonist on pro-inflammatory cytokine expression in EECs.

**Additional file 3: Figure S2.** Comparison of caloric intake of Tas1r3−/− and Tas1r3+/+ mice fed WD or ND for 10 weeks ad libitum.

**Additional file 4: Figure S3.** The effect of TAS1R3 deficiency on intestinal inflammation in a murine DSS-colitis model.

**Additional file 5: Figure S4.** Schematic depicting the roles of TAS1R3 in the intestinal inflammation.

**Additional file 6.** Uncropped western blot figures.

## Data Availability

The raw transcriptome datasets generated during the current study are available in the Gene Expression Omnibus (GEO) repository (accession numbers GSE140972 and GSE190059) and the raw gut microbiota datasets generated during the current study are available in the National Center for Biotechnology Information (NCBI) Sequence Read Archive (SRA) (accession number PRJNA784369) repositories. All data relevant to the study are included in the article or uploaded as online supplementary information.

## Abbreviations

AMP: Antimicrobial peptide
DSS: Dextran sulfate sodium
DEG: Differentially expressed gene
EEC: Enteroendocrine cell
FDR: False discovery rate
GLP-1: Glucagon-like peptide 1
GSEA: Gene set enrichment analysis
IBD: Inflammatory bowel disease
IL: Interleukin
MCP-1: Monocyte chemoattractant protein-1
ND: Normal diet
PCA: Principal component analysis
PCoA: Principal coordinate analysis
RNA-seq: RNA sequencing
TAS1R3: Taste receptor type 1 member 3
*Tas1r3*^+^^*/*^^+^: Wild-type littermate control
*Tas1r3*^*–/–*^: *Tas1r3*-Knockout
TJP: Tight-junction protein
TNFα: Tumor necrosis factor-alpha
TR: Taste receptor
WD: Western diet
