# Supplementary material for: Gut taste receptor type 1 member 3 is an intrinsic regulator of Western diet-induced intestinal inflammation
Source: BMC Med. 2023 Apr 28;21:165. doi: 10.1186/s12916-023-02848-0 (PMC10148556; doi:10.1186/s12916-023-02848-0)
Supplement: Supplementary file 1 — Additional file 1: Table S1. siRNA target sequences. Table S2. RT-PCR primers. Table S3. List of transcriptome datasets retrieved from publicly available Gene Expression Omnibus. Table S4. Descriptive statistics. [file 12916_2023_2848_MOESM1_ESM.docx]

**Table S1.** **siRNA target sequences.**

| **Target gene** | **Sense** | **Antisense** |
| --- | --- | --- |
| **Human *TAS1R3*** | CUGUCUACGCAGCUGUGUA | UACACAGCUGCGUAGACAG |

**Table S2. RT-PCR primers.**

| **Gene** | **Forward primer sequence**  **(5′–3′)** | **Reverse primer sequence**  **(5′–3′)** |
| --- | --- | --- |
| **Mouse *Gapdh*** | AGGTCGGTGTGAACGGATTTG | TGTAGACCATGTAGTTGAGGTCA |
| **Mouse *Tas1r3*** | TGCTGCCTACTGCAACTACAC | CCGGTCACTTAGCCGATCC |
| **Mouse *Il1b*** | CTGTGACTCATGGGATGATGATG | CGGAGCCTGTAGTGCAGTTG |
| **Mouse**  ***Il6*** | TAGTCCTTCCTACCCCAATTTCC | TTGGTCCTTAGCCACTCCTTC |
| **Mouse**  ***Tnf*** | CCCTCACACTCAGATCATCTTCT | GCTACGACGTGGGCTACAG |
| **Human *GAPDH*** | CCACTCCTCCACCTTTGACG | CCACCACCCTGTTGCTGTAG |
| **Human *TAS1R3*** | CACCAGACGTTCTCTGTCTACG | CTGAGGCGTTGCACTGAAGA |
| **Human *GCG*** | CTGAAGGGACCTTTACCAGTGA | CCTGGCGGCAAGATTATCAAG |
| **Human *IL1B*** | ATGATGGCTTATTACAGTGGCAA | GTCGGAGATTCGTAGCTGGA |
| **Human *IL6*** | CCTGAACCTTCCAAAGATGGC | TTCACCAGGCAAGTCTCCTCA |
| **Human**  ***IL8*** | ATGACTTCCAAGCTGGCCGTGGCT | TCTCAGCCCTCTTCAAAAACTTCTC |
| **Human *TNF*** | GAGGCCAAGCCCTGGTATG | CGGGCCGATTGATCTCAGC |
| **Human *CCL2***  **(*MCP1*)** | GCTCAGCCAGATGCAATCA | TTTGCTTGTCCAGGTGGTC |

**Table S3.** **List of transcriptome datasets retrieved from publicly available Gene Expression Omnibus.**

| **Study title** | **GEO**  **accession number** | **Reference** |
| --- | --- | --- |
| Integrated analysis of DNA methylation and gene expression profiles identified S100A9 as a potential biomarker in ulcerative colitis | GSE160804 | Su S et al., 2020 |
| Concordance between gene expression in peripheral whole blood and colonic tissue in children with inflammatory bowel disease | GSE126124 | Palmer NP et al., 2019 |
| Differential Gene Expression between involved and uninvolved sites of Crohn’s disease: Insights into a Distinctive Pathogenesis Profile | GSE95095 | Zhao X, 2019 |
| Mucosal gene expression profiling in patients with inflammatory bowel disease study | GSE75214 | Vancamelbeke M et al., 2017 |
| Differential Gene Expression between Active and Quiescent Stages of Ulcerative Colitis: Insights into a Distinctive Pathogenesis Profile | GSE53306 | Zhao X et al., 2015 |

**Table S4. Descriptive statistics.**

| **Variable** | **Group** | | | **Mean** | **Median** | **Min.** | **Max.** | **S.D.** | **Data** |
| --- | --- | --- | --- | --- | --- | --- | --- | --- | --- |
| Inflammation score | ND | | | 0 | 0 | 0 | 0 | 0 | Fig. 1b |
|  | WD | | | 5.77 | 6 | 2 | 8.3 | 1.976 |  |
| TAS1R3 protein expression (TAS1R3^+^ cells/field) | ND | | | 16.29 | 15.59 | 8.741 | 22.91 | 3.673 | Fig. 1g |
|  | WD | | | 23.96 | 22.98 | 19.4 | 30.77 | 4.245 |  |
| Transcript abundance of TAS1R3 (*TAS1R3/GAPDH*) | Control siRNA | | | 1 | 0.9915 | 0.9544 | 1.069 | 0.04971 | Fig. 2h |
|  | TAS1R3 siRNA | | | 0.3393 | 0.3684 | 0.24 | 0.4342 | 0.07931 |  |
| Active GLP-1 (7-36) Fold change over vehicle | Fru^+^Glu^+^Pal^+^ | | - | 1 | 1 | 0.9646 | 1.035 | 0.03537 | Fig. 2j |
|  | Con siRNA | | + |  |  |  |  |  |  |
|  | TAS1R3 siRNA | | - |  |  |  |  |  |  |
|  | Fru^+^Glu^+^Pal^+^ | | - | 0.91 | 0.993 | 0.727 | 1.01 | 0.1587 |  |
|  | Con siRNA | | - |  |  |  |  |  |  |
|  | TAS1R3 siRNA | | + |  |  |  |  |  |  |
|  | Fru^+^Glu^+^Pal^+^ | | + | 11.29 | 10.96 | 9.629 | 13.29 | 1.855 |  |
|  | Con siRNA | | + |  |  |  |  |  |  |
|  | TAS1R3 siRNA | | - |  |  |  |  |  |  |
|  | Fru^+^Glu^+^Pal^+^ | | + | 2.553 | 2.276 | 2.22 | 3.163 | 0.5292 |  |
|  | Con siRNA | | - |  |  |  |  |  |  |
|  | TAS1R3 siRNA | | + |  |  |  |  |  |  |
| IL-8 Fold-change over vehicle | Fru^+^Glu^+^Pal^+^ | | - | 1.045 | 1.015 | 0.9848 | 1.134 | 0.07897 | Fig. 2k |
|  | Con siRNA | | + |  |  |  |  |  |  |
|  | TAS1R3 siRNA | | - |  |  |  |  |  |  |
|  | Fru^+^Glu^+^Pal^+^ | | - | 0.6471 | 0.755 | 0.4152 | 0.771 | 0.201 |  |
|  | Con siRNA | | - |  |  |  |  |  |  |
|  | TAS1R3 siRNA | | + |  |  |  |  |  |  |
|  | Fru^+^Glu^+^Pal^+^ | | + | 5.849 | 5.714 | 5.131 | 6.703 | 0.7945 |  |
|  | Con siRNA | | + |  |  |  |  |  |  |
|  | TAS1R3 siRNA | | - |  |  |  |  |  |  |
|  | Fru^+^Glu^+^Pal^+^ | | + | 2.079 | 1.811 | 1.58 | 2.845 | 0.6737 |  |
|  | Con siRNA | | - |  |  |  |  |  |  |
|  | TAS1R3 siRNA | | + |  |  |  |  |  |  |
| TNF-α Fold-change over vehicle | Fru^+^Glu^+^Pal^+^ | | - | 1.045 | 1.015 | 0.9848 | 1.134 | 0.07897 | Fig. 2i |
|  | Con siRNA | | + |  |  |  |  |  |  |
|  | TAS1R3 siRNA | | - |  |  |  |  |  |  |
|  | Fru^+^Glu^+^Pal^+^ | | - | 0.5317 | 0.5115 | 0.3125 | 0.771 | 0.2299 |  |
|  | Con siRNA | | - |  |  |  |  |  |  |
|  | TAS1R3 siRNA | | + |  |  |  |  |  |  |
|  | Fru^+^Glu^+^Pal^+^ | | + | 4.314 | 4.525 | 3.714 | 4.703 | 0.5271 |  |
|  | Con siRNA | | + |  |  |  |  |  |  |
|  | TAS1R3 siRNA | | - |  |  |  |  |  |  |
|  | Fru^+^Glu^+^Pal^+^ | | + | 1.879 | 1.845 | 1.811 | 1.98 | 0.08912 |  |
|  | Con siRNA | | - |  |  |  |  |  |  |
|  | TAS1R3 siRNA | | + |  |  |  |  |  |  |
| Active GLP-1 (7-36) Fold change over vehicle | Fru^+^Glu^+^Pal^+^ | | - | 1 | 1 | 0.9741 | 1.026 | 0.02592 | Fig. 2m |
|  | TAS1R3 antagonist (Lactisole) | | - |  |  |  |  |  |  |
|  | Fru^+^Glu^+^Pal^+^ | | - | 0.8542 | 0.8542 | 0.7499 | 0.9586 | 0.1043 |  |
|  | TAS1R3 antagonist (Lactisole) | | + |  |  |  |  |  |  |
|  | Fru^+^Glu^+^Pal^+^ | | + | 6.797 | 6.797 | 6.698 | 6.897 | 0.1043 |  |
|  | TAS1R3 antagonist (Lactisole) | | - |  |  |  |  |  |  |
|  | Fru^+^Glu^+^Pal^+^ | | + | 2.728 | 2.728 | 2.313 | 3.143 | 0.4154 |  |
|  | TAS1R3 antagonist (Lactisole) | | + |  |  |  |  |  |  |
| IL-8 Fold-change over vehicle | Fru^+^Glu^+^Pal^+^ | | - | 1 | 1 | 0.9877 | 1.012 | 0.01227 | Fig. 2n |
|  | TAS1R3 antagonist (Lactisole) | | - |  |  |  |  |  |  |
|  | Fru^+^Glu^+^Pal^+^ | | - | 0.9885 | 0.9885 | 0.953 | 1.024 | 0.03557 |  |
|  | TAS1R3 antagonist (Lactisole) | | + |  |  |  |  |  |  |
|  | Fru^+^Glu^+^Pal^+^ | | + | 6.216 | 6.549 | 5.453 | 6.645 | 0.6619 |  |
|  | TAS1R3 antagonist (Lactisole) | | - |  |  |  |  |  |  |
|  | Fru^+^Glu^+^Pal^+^ | | + | 2.261 | 2.327 | 2.02 | 2.435 | 0.2156 |  |
|  | TAS1R3 antagonist (Lactisole) | | + |  |  |  |  |  |  |
| TNF-α Fold-change over vehicle | Fru^+^Glu^+^Pal^+^ | | - | 1 | 1 | 0.9741 | 1.026 | 0.02592 | Fig. 2o |
|  | TAS1R3 antagonist (Lactisole) | | - |  |  |  |  |  |  |
|  | Fru^+^Glu^+^Pal^+^ | | - | 0.8119 | 0.7596 | 0.7309 | 0.9453 | 0.1164 |  |
|  | TAS1R3 antagonist (Lactisole) | | + |  |  |  |  |  |  |
|  | Fru^+^Glu^+^Pal^+^ | | + | 3.417 | 3.417 | 3 | 3.834 | 0.4173 |  |
|  | TAS1R3 antagonist (Lactisole) | | - |  |  |  |  |  |  |
|  | Fru^+^Glu^+^Pal^+^ | | + | 1.208 | 1.208 | 1.152 | 1.264 | 0.0559 |  |
|  | TAS1R3 antagonist (Lactisole) | | + |  |  |  |  |  |  |
| Transcript abundance of *Tas1r3* (*Tas1r3*/*Gapdh*) | *Tas1r3*^+/+^ ND | | | 0.005684 | 0.005602 | 0.004099 | 0.007539 | 0.00116 | Fig. 3a |
|  | *Tas1r3*^-/-^ ND | | | 2.59e-005 | 1.361e-005 | 1.3e-006 | 9.137e-005 | 3.042e-005 |  |
|  | *Tas1r3*^+/+^ WD | | | 0.02351 | 0.02389 | 0.01454 | 0.03586 | 0.00636 |  |
|  | *Tas1r3*^-/-^ WD | | | 2.59e-005 | 1.361e-005 | 1.3e-006 | 9.137e-005 | 3.042e-005 |  |
| TAS1R3 protein expression (TAS1R3^+^ cells/field) | *Tas1r3*^+/+^ ND | | | 16.36 | 16.06 | 14.39 | 19.36 | 1.366 | Fig. 3b |
|  | *Tas1r3*^-/-^ ND | | | 0.4917 | 0.3085 | 0.112 | 1.875 | 0.5216 |  |
|  | *Tas1r3*^+/+^ WD | | | 23.26 | 23.21 | 18.98 | 27.58 | 2.554 |  |
|  | *Tas1r3*^-/-^ WD | | | 1.15 | 0.7805 | 0.112 | 3.32 | 1.077 |  |
| Spleen weight (g) | *Tas1r3*^+/+^ ND | | | 0.0892 | 0.089 | 0.025 | 0.148 | 0.0391 | Fig. 3c |
|  | *Tas1r3*^-/-^ ND | | | 0.0961 | 0.0795 | 0.058 | 0.194 | 0.04334 |  |
|  | *Tas1r3*^+/+^ WD | | | 0.2085 | 0.1925 | 0.127 | 0.31 | 0.06195 |  |
|  | *Tas1r3*^-/-^ WD | | | 0.0931 | 0.0745 | 0.058 | 0.194 | 0.04192 |  |
| Small intestine length (cm) | *Tas1r3*^+/+^ ND | | | 43.71 | 43.85 | 40.5 | 47 | 1.895 | Fig. 3d |
|  | *Tas1r3*^-/-^ ND | | | 42.11 | 42.3 | 36 | 47.3 | 3.413 |  |
|  | *Tas1r3*^+/+^ WD | | | 32 | 32.5 | 27 | 36 | 2.759 |  |
|  | *Tas1r3*^-/-^ WD | | | 39.34 | 39.25 | 36 | 42.5 | 2.017 |  |
| Colon length (cm) | *Tas1r3*^+/+^ ND | | | 7.3 | 7.3 | 6.5 | 7.9 | 0.3944 | Fig. 3e |
|  | *Tas1r3*^-/-^ ND | | | 7.66 | 7.65 | 7.1 | 8.3 | 0.3565 |  |
|  | *Tas1r3*^+/+^ WD | | | 5.21 | 5.15 | 4.7 | 6.3 | 0.5021 |  |
|  | *Tas1r3*^-/-^ WD | | | 6.7 | 6.75 | 6.2 | 7.2 | 0.3621 |  |
| Inflammation score | *Tas1r3*^+/+^ ND | | | 0 | 0 | 0 | 0 | 0 | Fig. 3g |
|  | *Tas1r3*^-/-^ ND | | | 0 | 0 | 0 | 0 | 0 |  |
|  | *Tas1r3*^+/+^ WD | | | 5.55 | 5.35 | 3.3 | 8 | 1.445 |  |
|  | *Tas1r3*^-/-^ WD | | | 0.74 | 0 | 0 | 2.1 | 0.3096 |  |
| Transcript abundance of *Il1b* (*Il1b/Gapdh*) | *Tas1r3*^+/+^ ND | | | 0.1429 | 0.1292 | 0.09915 | 0.1929 | 0.03775 | Fig. 3h |
|  | *Tas1r3*^-/-^ ND | | | 0.125 | 0.1144 | 0.03937 | 0.1875 | 0.04979 |  |
|  | *Tas1r3*^+/+^ WD | | | 1.984 | 1.875 | 0.3988 | 3.527 | 0.8728 |  |
|  | *Tas1r3*^-/-^ WD | | | 0.2965 | 0.1986 | 0.07387 | 0.9262 | 0.2784 |  |
| Transcript abundance of *Tnfa* (*Tnfa* /*Gapdh*) | *Tas1r3*^+/+^ ND | | | 0.1429 | 0.08489 | 0.01712 | 0.2777 | 0.1046 | Fig. 3i |
|  | *Tas1r3*^-/-^ ND | | | 0.1897 | 0.1141 | 0.09444 | 0.3924 | 0.05228 |  |
|  | *Tas1r3*^+/+^ WD | | | 0.1897 | 0.1141 | 0.3183 | 0.7719 | 0.1383 |  |
|  | *Tas1r3*^-/-^ WD | | | 0.1267 | 0.09467 | 0.04475 | 0.3924 | 0.1082 |  |
| Transcript abundance of *Il6* (*Il6* /*Gapdh*) | *Tas1r3*^+/+^ ND | | | 0.1429 | 0.1442 | 0.06891 | 0.2596 | 0.0647 | Fig. 3j |
|  | *Tas1r3*^-/-^ ND | | | 0.1523 | 0.1147 | 0.03845 | 0.2917 | 0.09538 |  |
|  | *Tas1r3*^+/+^ WD | | | 0.7925 | 0.7366 | 0.441 | 1.223 | 0.3063 |  |
|  | *Tas1r3*^-/-^ WD | | | 0.2673 | 0.2646 | 0.1773 | 0.3466 | 0.06031 |  |
| CD45^+^ cells/field | *Tas1r3*^+/+^ ND | | | 2.431 | 1.562 | 0.065 | 8.992 | 3.267 | Fig. 3k |
|  | *Tas1r3*^-/-^ ND | | | 2.194 | 1.41 | 0.114 | 4.321 | 1.734 |  |
|  | *Tas1r3*^+/+^ WD | | | 33.45 | 33.36 | 25.73 | 45 | 6.015 |  |
|  | *Tas1r3*^-/-^ WD | | | 11.27 | 11.51 | 5 | 15.49 | 3.315 |  |
| CD4^+^ T cells/field | *Tas1r3*^+/+^ ND | | | 2.908 | 2.9 | 1.48 | 4.1 | 0.8835 | Fig. 3i |
|  | *Tas1r3*^-/-^ ND | | | 2.88 | 3 | 1.5 | 4.1 | 0.9028 |  |
|  | *Tas1r3*^+/+^ WD | | | 8.73 | 8.4 | 5.4 | 13 | 2.132 |  |
|  | *Tas1r3*^-/-^ WD | | | 5.7 | 6.2 | 3.7 | 7.3 | 1.398 |  |
| CD8^+^ T cells/field | *Tas1r3*^+/+^ ND | | | 0.86 | 0.842 | 0.065 | 1.992 | 0.788 | Fig. 3m |
|  | *Tas1r3*^-/-^ ND | | | 1.051 | 1.15 | 0.114 | 1.472 | 0.4649 |  |
|  | *Tas1r3*^+/+^ WD | | | 7.142 | 6.55 | 4.856 | 10.87 | 1.905 |  |
|  | *Tas1r3*^-/-^ WD | | | 3.408 | 3.444 | 1.669 | 4.784 | 0.9637 |  |
| CD11b^+^ dendritic cells/field | *Tas1r3*^+/+^ ND | | | 0.86 | 0.842 | 0.065 | 1.992 | 0.788 | Fig. 3n |
|  | *Tas1r3*^-/-^ ND | | | 1.051 | 1.15 | 0.114 | 1.472 | 0.4649 |  |
|  | *Tas1r3*^+/+^ WD | | | 5.192 | 5.216 | 3.4 | 6.7 | 1.042 |  |
|  | *Tas1r3*^-/-^ WD | | | 2.208 | 2.261 | 1.287 | 2.9 | 0.6077 |  |
| *Lipin1* expression RPKM | *Tas1r3*^+/+^ WD | | | 0.3299 | 0.3157 | 0.1179 | 0.6047 | 0.1727 | Fig. 4h |
|  | *Tas1r3*^-/-^ WD | | | 1.312 | 1.293 | 1.198 | 1.465 | 0.1152 |  |
| *Pparg* expression RPKM | *Tas1r3*^+/+^ WD | | | 0.2764 | 0.2272 | 0.1879 | 0.4809 | 0.1118 | Fig. 4i |
|  | *Tas1r3*^-/-^ WD | | | 2.37 | 2.079 | 1.366 | 3.676 | 0.9101 |  |
| p-mTOR-^ser2448^ protein expression level (normalized to α-tubulin) | *Tas1r3*^+/+^ ND | | | 0.1931 | 0.2052 | 0.1052 | 0.2809 | 0.0661 | Fig. 5b |
|  | *Tas1r3*^-/-^ ND | | | 0.05493 | 0.04778 | 0.03353 | 0.09112 | 0.02339 |  |
|  | *Tas1r3*^+/+^ WD | | | 0.3018 | 0.3146 | 0.1814 | 0.4035 | 0.07735 |  |
|  | *Tas1r3*^-/-^ WD | | | 0.1354 | 0.1352 | 0.0889 | 0.1812 | 0.04577 |  |
| PPARγ protein expression level (normalized to α-tubulin) | *Tas1r3*^+/+^ ND | | | 0.6851 | 0.6886 | 0.4814 | 0.9035 | 0.147 | Fig. 5c |
|  | *Tas1r3*^-/-^ ND | | | 2.913 | 2.91 | 2.366 | 3.376 | 0.4171 |  |
|  | *Tas1r3*^+/+^ WD | | | 0.2764 | 0.2272 | 0.1879 | 0.4809 | 0.1118 |  |
|  | *Tas1r3*^-/-^ WD | | | 2.596 | 2.631 | 2.112 | 2.966 | 0.3846 |  |
| Transcript abundance of MTOR (*MTOR/GAPDH*) | Fru^+^Glu^+^Pal^+^ | | - | 1 | 1.031 | 0.7433 | 1.167 | 0.1735 | Fig. 5d |
|  | Con siRNA | | + |  |  |  |  |  |  |
|  | TAS1R3 siRNA | | - |  |  |  |  |  |  |
|  | Fru^+^Glu^+^Pal^+^ | | - | 0.8815 | 0.8953 | 0.8306 | 0.9125 | 0.03219 |  |
|  | Con siRNA | | - |  |  |  |  |  |  |
|  | TAS1R3 siRNA | | + |  |  |  |  |  |  |
|  | Fru^+^Glu^+^Pal^+^ | | + | 1 | 0.9623 | 0.8027 | 1.298 | 0.1967 |  |
|  | Con siRNA | | + |  |  |  |  |  |  |
|  | TAS1R3 siRNA | | - |  |  |  |  |  |  |
|  | Fru^+^Glu^+^Pal^+^ | | + | 0.3352 | 0.3144 | 0.2804 | 0.3948 | 0.05346 |  |
|  | Con siRNA | | - |  |  |  |  |  |  |
|  | TAS1R3 siRNA | | + |  |  |  |  |  |  |
| Transcript abundance of PPARG (*PPARG /GAPDH*) | Fru^+^Glu^+^Pal^+^ | | - | 1 | 0.9666 | 0.9492 | 1.082 | 0.05961 | Fig. 5e |
|  | Con siRNA | | + |  |  |  |  |  |  |
|  | TAS1R3 siRNA | | - |  |  |  |  |  |  |
|  | Fru^+^Glu^+^Pal^+^ | | - | 2.425 | 2.296 | 2.158 | 2.921 | 0.3062 |  |
|  | Con siRNA | | - |  |  |  |  |  |  |
|  | TAS1R3 siRNA | | + |  |  |  |  |  |  |
|  | Fru^+^Glu^+^Pal^+^ | | + | 0.6654 | 0.6075 | 0.3332 | 0.9896 | 0.2447 |  |
|  | Con siRNA | | + |  |  |  |  |  |  |
|  | TAS1R3 siRNA | | - |  |  |  |  |  |  |
|  | Fru^+^Glu^+^Pal^+^ | | + | 1.868 | 1.947 | 1.631 | 1.976 | 0.1457 |  |
|  | Con siRNA | | - |  |  |  |  |  |  |
|  | TAS1R3 siRNA | | + |  |  |  |  |  |  |
| PPARγ protein expression level (normalized to α-tubulin) | Fru^+^Glu^+^Pal^+^ | | - | 0.1931 | 0.2052 | 0.1052 | 0.2809 | 0.0661 | Fig. 5f |
|  | Con siRNA | | + |  |  |  |  |  |  |
|  | TAS1R3 siRNA | | - |  |  |  |  |  |  |
|  | Fru^+^Glu^+^Pal^+^ | | - | 1.212 | 1.121 | 0.8116 | 1.957 | 0.4266 |  |
|  | Con siRNA | | - |  |  |  |  |  |  |
|  | TAS1R3 siRNA | | + |  |  |  |  |  |  |
|  | Fru^+^Glu^+^Pal^+^ | | + | 0.05493 | 0.04778 | 0.03353 | 0.09112 | 0.02339 |  |
|  | Con siRNA | | + |  |  |  |  |  |  |
|  | TAS1R3 siRNA | | - |  |  |  |  |  |  |
|  | Fru^+^Glu^+^Pal^+^ | | + | 0.3518 | 0.3386 | 0.2541 | 0.5035 | 0.08912 |  |
|  | Con siRNA | | - |  |  |  |  |  |  |
|  | TAS1R3 siRNA | | + |  |  |  |  |  |  |
| Transcript abundance of MTOR (*MTOR/GAPDH*) | Fru^+^Glu^+^Pal^+^ | | - | 1.1 | 0.9993 | 0.8198 | 1.678 | 0.3345 | Fig. 5g |
|  | TAS1R3 antagonist (Lactisole) | | - |  |  |  |  |  |  |
|  | Fru^+^Glu^+^Pal^+^ | | - | 0.8373 | 0.8778 | 0.7023 | 0.9094 | 0.08565 |  |
|  | TAS1R3 antagonist (Lactisole) | | + |  |  |  |  |  |  |
|  | Fru^+^Glu^+^Pal^+^ | | + | 6.845 | 7.134 | 5.934 | 7.6 | 0.6735 |  |
|  | TAS1R3 antagonist (Lactisole) | | - |  |  |  |  |  |  |
|  | Fru^+^Glu^+^Pal^+^ | | + | 2.548 | 2.401 | 1.957 | 3.221 | 0.5076 |  |
|  | TAS1R3 antagonist (Lactisole) | | + |  |  |  |  |  |  |
| Transcript abundance of PPARG (*PPARG /GAPDH*) | Fru^+^Glu^+^Pal^+^ | | - | 1.086 | 1.133 | 0.7085 | 1.46 | 0.3099 | Fig. 5h |
|  | TAS1R3 antagonist (Lactisole) | | - |  |  |  |  |  |  |
|  | Fru^+^Glu^+^Pal^+^ | | - | 2.613 | 2.655 | 2.28 | 2.865 | 0.2167 |  |
|  | TAS1R3 antagonist (Lactisole) | | + |  |  |  |  |  |  |
|  | Fru^+^Glu^+^Pal^+^ | | + | 0.4637 | 0.4666 | 0.3322 | 0.5761 | 0.08687 |  |
|  | TAS1R3 antagonist (Lactisole) | | - |  |  |  |  |  |  |
|  | Fru^+^Glu^+^Pal^+^ | | + | 2.051 | 2.231 | 1.668 | 2.325 | 0.3023 |  |
|  | TAS1R3 antagonist (Lactisole) | | + |  |  |  |  |  |  |
| PPARγ protein expression level (normalized to α-tubulin) | Fru^+^Glu^+^Pal^+^ | | - | 0.8708 | 0.8709 | 0.7284 | 1.008 | 0.09171 | Fig. 5i |
|  | TAS1R3 antagonist (Lactisole) | | - |  |  |  |  |  |  |
|  | Fru^+^Glu^+^Pal^+^ | | - | 3.47 | 3.386 | 2.945 | 4.035 | 0.3944 |  |
|  | TAS1R3 antagonist (Lactisole) | | + |  |  |  |  |  |  |
|  | Fru^+^Glu^+^Pal^+^ | | + | 0.4872 | 0.3889 | 0.2889 | 0.7616 | 0.2069 |  |
|  | TAS1R3 antagonist (Lactisole) | | - |  |  |  |  |  |  |
|  | Fru^+^Glu^+^Pal^+^ | | + | 2.212 | 2.138 | 1.879 | 2.809 | 0.3337 |  |
|  | TAS1R3 antagonist (Lactisole) | | + |  |  |  |  |  |  |
| Transcript abundance of TNF (*TNF/GAPDH*) | Fru^+^Glu^+^Pal^+^ | | + | 1 | 0.8658 | 0.6707 | 1.481 | 0.3516 | Fig. 5j |
|  | Con siRNA | | + |  |  |  |  |  |  |
|  | TAS1R3 siRNA | | - |  |  |  |  |  |  |
|  | PPARγ antagonist (GW9662) | | - |  |  |  |  |  |  |
|  | Fru^+^Glu^+^Pal^+^ | | + | 0.1267 | 0.1123 | 0.09616 | 0.1956 | 0.0396 |  |
|  | Con siRNA | | - |  |  |  |  |  |  |
|  | TAS1R3 siRNA | | + |  |  |  |  |  |  |
|  | PPARγ antagonist (GW9662) | | - |  |  |  |  |  |  |
|  | Fru^+^Glu^+^Pal^+^ | | + | 0.7119 | 0.6856 | 0.6218 | 0.8092 | 0.07742 |  |
|  | Con siRNA | | + |  |  |  |  |  |  |
|  | TAS1R3 siRNA | | - |  |  |  |  |  |  |
|  | PPARγ antagonist (GW9662) | | + |  |  |  |  |  |  |
|  | Fru^+^Glu^+^Pal^+^ | | + | 0.7661 | 0.755 | 0.6121 | 0.9206 | 0.1181 |  |
|  | Con siRNA | | - |  |  |  |  |  |  |
|  | TAS1R3 siRNA | | + |  |  |  |  |  |  |
|  | PPARγ antagonist (GW9662) | | + |  |  |  |  |  |  |
| Transcript abundance of IL8 (*IL8/GAPDH*) | Fru^+^Glu^+^Pal^+^ | | + | 1 | 0.9684 | 0.7307 | 1.243 | 0.1967 | Fig. 5k |
|  | Con siRNA | | + |  |  |  |  |  |  |
|  | TAS1R3 siRNA | | - |  |  |  |  |  |  |
|  | PPARγ antagonist (GW9662) | | - |  |  |  |  |  |  |
|  | Fru^+^Glu^+^Pal^+^ | | + | 0.0621 | 0.06425 | 0.04731 | 0.06869 | 0.008494 |  |
|  | Con siRNA | | - |  |  |  |  |  |  |
|  | TAS1R3 siRNA | | + |  |  |  |  |  |  |
|  | PPARγ antagonist (GW9662) | | - |  |  |  |  |  |  |
|  | Fru^+^Glu^+^Pal^+^ | | + | 0.7266 | 0.6712 | 0.4939 | 0.9583 | 0.2101 |  |
|  | Con siRNA | | + |  |  |  |  |  |  |
|  | TAS1R3 siRNA | | - |  |  |  |  |  |  |
|  | PPARγ antagonist (GW9662) | | + |  |  |  |  |  |  |
|  | Fru^+^Glu^+^Pal^+^ | | + | 0.4843 | 0.4737 | 0.3334 | 0.6914 | 0.1313 |  |
|  | Con siRNA | | - |  |  |  |  |  |  |
|  | TAS1R3 siRNA | | + |  |  |  |  |  |  |
|  | PPARγ antagonist (GW9662) | | + |  |  |  |  |  |  |
| TNF-α Fold-change over vehicle | Fru^+^Glu^+^Pal^+^ | | + | 1 | 1.049 | 0.8609 | 1.09 | 0.1222 | Fig. 5l |
|  | Con siRNA | | + |  |  |  |  |  |  |
|  | TAS1R3 siRNA | | - |  |  |  |  |  |  |
|  | PPARγ antagonist (GW9662) | | - |  |  |  |  |  |  |
|  | Fru^+^Glu^+^Pal^+^ | | + | 0.4355 | 0.4277 | 0.4199 | 0.459 | 0.02066 |  |
|  | Con siRNA | | - |  |  |  |  |  |  |
|  | TAS1R3 siRNA | | + |  |  |  |  |  |  |
|  | PPARγ antagonist (GW9662) | | - |  |  |  |  |  |  |
|  | Fru^+^Glu^+^Pal^+^ | | + | 0.9641 | 0.9641 | 0.7698 | 1.158 | 0.1943 |  |
|  | Con siRNA | | + |  |  |  |  |  |  |
|  | TAS1R3 siRNA | | - |  |  |  |  |  |  |
|  | PPARγ antagonist (GW9662) | | + |  |  |  |  |  |  |
|  | Fru^+^Glu^+^Pal^+^ | | + | 0.7253 | 0.7253 | 0.7043 | 0.7462 | 0.02093 |  |
|  | Con siRNA | | - |  |  |  |  |  |  |
|  | TAS1R3 siRNA | | + |  |  |  |  |  |  |
|  | PPARγ antagonist (GW9662) | | + |  |  |  |  |  |  |
| IL-8 Fold-change over vehicle | Fru^+^Glu^+^Pal^+^ | | + | 1 | 0.9769 | 0.8772 | 1.146 | 0.1358 | Fig. 5m |
|  | Con siRNA | | + |  |  |  |  |  |  |
|  | TAS1R3 siRNA | | - |  |  |  |  |  |  |
|  | PPARγ antagonist (GW9662) | | - |  |  |  |  |  |  |
|  | Fru^+^Glu^+^Pal^+^ | | + | 0.3554 | 0.3097 | 0.2701 | 0.4864 | 0.1152 |  |
|  | Con siRNA | | - |  |  |  |  |  |  |
|  | TAS1R3 siRNA | | + |  |  |  |  |  |  |
|  | PPARγ antagonist (GW9662) | | - |  |  |  |  |  |  |
|  | Fru^+^Glu^+^Pal^+^ | | + | 0.9142 | 0.9142 | 0.8793 | 0.9491 | 0.03489 |  |
|  | Con siRNA | | + |  |  |  |  |  |  |
|  | TAS1R3 siRNA | | - |  |  |  |  |  |  |
|  | PPARγ antagonist (GW9662) | | + |  |  |  |  |  |  |
|  | Fru^+^Glu^+^Pal^+^ | | + | 0.812 | 0.812 | 0.7696 | 0.8544 | 0.04236 |  |
|  | Con siRNA | | - |  |  |  |  |  |  |
|  | TAS1R3 siRNA | | + |  |  |  |  |  |  |
|  | PPARγ antagonist (GW9662) | | + |  |  |  |  |  |  |
| Relative *Tjp3* expression | *Tas1r3*^+/+^ ND | | | 1 | 1.049 | 0.5593 | 1.252 | 0.2491 | Fig. 6a |
|  | *Tas1r3*^-/-^ ND | | | 1.122 | 1.099 | 0.9743 | 1.338 | 0.1219 |  |
|  | *Tas1r3*^+/+^ WD | | | 0.7147 | 0.7486 | 0.4362 | 0.9045 | 0.1664 |  |
|  | *Tas1r3*^-/-^ WD | | | 1.259 | 1.248 | 1.183 | 1.367 | 0.06153 |  |
| Relative *Ocln* expression | *Tas1r3*^+/+^ ND | | | 1 | 0.7992 | 0.5482 | 2.001 | 0.5172 | Fig. 6a |
|  | *Tas1r3*^-/-^ ND | | | 0.811 | 0.7448 | 0.513 | 1.23 | 0.3084 |  |
|  | *Tas1r3*^+/+^ WD | | | 0.346 | 0.2366 | 0.03687 | 1 | 0.3662 |  |
|  | *Tas1r3*^-/-^ WD | | | 4.721 | 4.6 | 4.505 | 5.337 | 0.3135 |  |
| Relative *Cldn1* expression | *Tas1r3*^+/+^ ND | | | 1 | 0.9361 | 0.585 | 1.607 | 0.3421 | Fig. 6a |
|  | *Tas1r3*^-/-^ ND | | | 1.589 | 1.512 | 0.5245 | 2.5 | 0.677 |  |
|  | *Tas1r3*^+/+^ WD | | | 0.2293 | 0.1854 | 0.135 | 0.496 | 0.1341 |  |
|  | *Tas1r3*^-/-^ WD | | | 1.468 | 1.509 | 0.9374 | 2.08 | 0.4028 |  |
| Relative *Cldn7* expression | *Tas1r3*^+/+^ ND | | | 1 | 0.9852 | 0.7487 | 1.231 | 0.1612 | Fig. 6a |
|  | *Tas1r3*^-/-^ ND | | | 1.253 | 1.257 | 1.209 | 1.283 | 0.02464 |  |
|  | *Tas1r3*^+/+^ WD | | | 0.5675 | 0.5772 | 0.4593 | 0.6565 | 0.08298 |  |
|  | *Tas1r3*^-/-^ WD | | | 0.8549 | 0.7684 | 0.6629 | 1.194 | 0.2031 |  |
| Relative *Reg3g* expression | *Tas1r3*^+/+^ ND | | | 1 | 1.073 | 0.6009 | 1.219 | 0.22 | Fig. 6b |
|  | *Tas1r3*^-/-^ ND | | | 1.788 | 1.688 | 0.9342 | 2.7 | 0.6219 |  |
|  | *Tas1r3*^+/+^ WD | | | 0.432 | 0.3572 | 0.1872 | 0.8923 | 0.2557 |  |
|  | *Tas1r3*^-/-^ WD | | | 1.304 | 1.393 | 0.4563 | 1.884 | 0.4779 |  |
| Relative *Lyz1* expression | *Tas1r3*^+/+^ ND | | | 1 | 1.089 | 0.5041 | 1.452 | 0.3984 | Fig. 6b |
|  | *Tas1r3*^-/-^ ND | | | 1.249 | 1.263 | 1.119 | 1.338 | 0.07332 |  |
|  | *Tas1r3*^+/+^ WD | | | 0.6022 | 0.6283 | 0.3401 | 0.7534 | 0.1612 |  |
|  | *Tas1r3*^-/-^ WD | | | 1.365 | 1.355 | 0.8763 | 1.684 | 0.2839 |  |
| Relative *Defa2* expression | *Tas1r3*^+/+^ ND | | | 1.05 | 1.086 | 0.4907 | 1.529 | 0.507 | Fig. 6b |
|  | *Tas1r3*^-/-^ ND | | | 1.405 | 1.324 | 0.9055 | 1.853 | 0.3501 |  |
|  | *Tas1r3*^+/+^ WD | | | 0.4821 | 0.4602 | 0.3296 | 0.7482 | 0.1584 |  |
|  | *Tas1r3*^-/-^ WD | | | 1.885 | 1.995 | 0.848 | 2.595 | 0.5851 |  |
| Relative *Defa3* expression | *Tas1r3*^+/+^ ND | | | 1.083 | 1.063 | 0.6712 | 1.544 | 0.4268 | Fig. 6b |
|  | *Tas1r3*^-/-^ ND | | | 2.298 | 2.374 | 1.581 | 2.789 | 0.4043 |  |
|  | *Tas1r3*^+/+^ WD | | | 0.5646 | 0.5498 | 0.3759 | 0.7484 | 0.1591 |  |
|  | *Tas1r3*^-/-^ WD | | | 1.333 | 1.457 | 0.6768 | 1.595 | 0.3586 |  |
| Occludin protein expression level (normalized to α-tubulin) | *Tas1r3*^+/+^ ND | | | 0.4685 | 0.4678 | 0.3348 | 0.5945 | 0.08487 | Fig. 6c |
|  | *Tas1r3*^-/-^ ND | | | 0.549 | 0.5573 | 0.3091 | 0.7284 | 0.151 |  |
|  | *Tas1r3*^+/+^ WD | | | 0.1764 | 0.1844 | 0.1299 | 0.2225 | 0.03806 |  |
|  | *Tas1r3*^-/-^ WD | | | 0.9373 | 0.9474 | 0.7616 | 1.112 | 0.1139 |  |
| Claudin-1 protein expression level (normalized to α-tubulin) | *Tas1r3*^+/+^ ND | | | 0.8685 | 0.8678 | 0.7945 | 0.9348 | 0.04932 | Fig. 6d |
|  | *Tas1r3*^-/-^ ND | | | 1.966 | 1.971 | 1.579 | 2.309 | 0.2815 |  |
|  | *Tas1r3*^+/+^ WD | | | 0.07743 | 0.02605 | 0.0132 | 0.1979 | 0.08705 |  |
|  | *Tas1r3*^-/-^ WD | | | 1.154 | 1.2 | 0.9568 | 1.312 | 0.1576 |  |
| Shannons’s diversity index | *Tas1r3*^+/+^ ND | | | 5.717 | 5.7 | 5 | 6.4 | 0.4875 | Fig. 6e |
|  | *Tas1r3*^-/-^ ND | | | 5.267 | 5.25 | 5 | 5.5 | 0.1751 |  |
|  | *Tas1r3*^+/+^ WD | | | 4.183 | 4.15 | 3.7 | 4.8 | 0.4167 |  |
|  | *Tas1r3*^-/-^ WD | | | 5.267 | 5.3 | 4.9 | 5.6 | 0.3011 |  |
| Faith’s phylogenetic index | *Tas1r3*^+/+^ ND | | | 19.75 | 19.5 | 18 | 22 | 1.541 | Fig. 6f |
|  | *Tas1r3*^-/-^ ND | | | 17.67 | 17.75 | 16 | 19 | 1.08 |  |
|  | *Tas1r3*^+/+^ WD | | | 11 | 10.75 | 9 | 14 | 1.817 |  |
|  | *Tas1r3*^-/-^ WD | | | 15.35 | 15.25 | 14.1 | 17 | 1.056 |  |
| *g_Butyrivibrio* normalized abundance | *Tas1r3*^+/+^ WD | | | -1.357 | -1 | -3 | -1 | 0.7449 | Fig. 6k |
|  | *Tas1r3*^-/-^ WD | | | 2.286 | 2 | 1 | 5 | 1.139 |  |
| *g_Roseburia* normalized abundance | *Tas1r3*^+/+^ WD | | | -1.967 | -1.966 | -4.42 | 0.5254 | 1.348 | Fig. 6k |
|  | *Tas1r3*^-/-^ WD | | | 1.73 | 1.406 | 0.3898 | 3.878 | 0.9361 |  |
| *g_Ruminococcus* normalized abundance | *Tas1r3*^+/+^ WD | | | -0.1429 | 0 | -1 | 0 | 0.3631 | Fig. 6k |
|  | *Tas1r3*^-/-^ WD | | | 0.5714 | 0 | 0 | 2 | 0.7559 |  |
| *g_Butyricicoccus* normalized abundance | *Tas1r3*^+/+^ WD | | | -0.7143 | -1 | -3 | 1 | 0.9139 | Fig. 6k |
|  | *Tas1r3*^-/-^ WD | | | 0.6429 | 1 | -1 | 2 | 0.8419 |  |
| *g_Faecalibacterium* normalized abundance | *Tas1r3*^+/+^ WD | | | -1.506 | -1.45 | -3.3 | -0.21 | 0.915 | Fig. 6k |
|  | *Tas1r3*^-/-^ WD | | | 0.4379 | 0.33 | 0.06 | 1.12 | 0.3279 |  |
| Butyrate μmol/g fecal contents | *Tas1r3*^+/+^ WD | | | 168.2 | 171.8 | 69 | 272.4 | 47.4 | Fig. 6l |
|  | *Tas1r3*^-/-^ WD | | | 269.1 | 285.2 | 107.9 | 375.6 | 82.26 |  |
| Intestinal *TAS1R3* expression | Non-IBD | | | 6.953 | 6.886 | 6.115 | 10.47 | 0.5738 | Fig. 8d |
|  | IBD | | | 7.494 | 7.11 | 5.424 | 13.59 | 1.149 |  |
| Intestinal *MTOR* expression | Non-IBD | | | 8.322 | 8.31 | 7.39 | 9.164 | 0.4688 | Fig. 8e |
|  | IBD | | | 8.64 | 8.871 | 7.511 | 9.351 | 0.5169 |  |
| Intestinal *PPARG* expression | Non-IBD | | | 9.929 | 10.11 | 7.39 | 11.86 | 1.076 | Fig. 8f |
|  | IBD | | | 7.919 | 7.97 | 5.231 | 11.64 | 0.894 |  |
| Transcript abundance of GCG (*GCG/GAPDH*) | Fru^+^Glu^+^Pal^+^ | | - | 1 | 0.9576 | 0.9266 | 1.193 | 0.11 | Additional File 2: Fig. S1d |
|  | Con siRNA | | + |  |  |  |  |  |  |
|  | TAS1R3 siRNA | | - |  |  |  |  |  |  |
|  | Fru^+^Glu^+^Pal^+^ | | - | 0.4688 | 0.4466 | 0.4347 | 0.5397 | 0.04463 |  |
|  | Con siRNA | | - |  |  |  |  |  |  |
|  | TAS1R3 siRNA | | + |  |  |  |  |  |  |
|  | Fru^+^Glu^+^Pal^+^ | | + | 4.015 | 4.105 | 3.105 | 4.541 | 0.5405 |  |
|  | Con siRNA | | + |  |  |  |  |  |  |
|  | TAS1R3 siRNA | | - |  |  |  |  |  |  |
|  | Fru^+^Glu^+^Pal^+^ | | + | 0.5245 | 0.5192 | 0.4719 | 0.5701 | 0.03619 |  |
|  | Con siRNA | | - |  |  |  |  |  |  |
|  | TAS1R3 siRNA | | + |  |  |  |  |  |  |
| Transcript abundance of IL1B (*IL1B /GAPDH*) | Fru^+^Glu^+^Pal^+^ | | - | 1 | 1.14 | 0.4625 | 1.386 | 0.3822 | Additional File 2: Fig. S1e |
|  | Con siRNA | | + |  |  |  |  |  |  |
|  | TAS1R3 siRNA | | - |  |  |  |  |  |  |
|  | Fru^+^Glu^+^Pal^+^ | | - | 0.6576 | 0.6494 | 0.3996 | 0.9897 | 0.2539 |  |
|  | Con siRNA | | - |  |  |  |  |  |  |
|  | TAS1R3 siRNA | | + |  |  |  |  |  |  |
|  | Fru^+^Glu^+^Pal^+^ | | + | 3.851 | 4.235 | 2.77 | 4.609 | 0.823 |  |
|  | Con siRNA | | + |  |  |  |  |  |  |
|  | TAS1R3 siRNA | | - |  |  |  |  |  |  |
|  | Fru^+^Glu^+^Pal^+^ | | + | 1.621 | 1.465 | 1.078 | 2.289 | 0.4589 |  |
|  | Con siRNA | | - |  |  |  |  |  |  |
|  | TAS1R3 siRNA | | + |  |  |  |  |  |  |
| Transcript abundance of IL6 (*IL6/GAPDH*) | Fru^+^Glu^+^Pal^+^ | | - | 1 | 1.031 | 0.8696 | 1.036 | 0.07294 | Additional File 2: Fig. S1f |
|  | Con siRNA | | + |  |  |  |  |  |  |
|  | TAS1R3 siRNA | | - |  |  |  |  |  |  |
|  | Fru^+^Glu^+^Pal^+^ | | - | 0.7853 | 0.7899 | 0.695 | 0.8698 | 0.07672 |  |
|  | Con siRNA | | - |  |  |  |  |  |  |
|  | TAS1R3 siRNA | | + |  |  |  |  |  |  |
|  | Fru^+^Glu^+^Pal^+^ | | + | 2.953 | 2.606 | 1.873 | 4.115 | 0.9715 |  |
|  | Con siRNA | | + |  |  |  |  |  |  |
|  | TAS1R3 siRNA | | - |  |  |  |  |  |  |
|  | Fru^+^Glu^+^Pal^+^ | | + | 1.084 | 0.9694 | 0.6523 | 1.44 | 0.3286 |  |
|  | Con siRNA | | - |  |  |  |  |  |  |
|  | TAS1R3 siRNA | | + |  |  |  |  |  |  |
| Transcript abundance of IL8 *(IL8/GAPDH*) | Fru^+^Glu^+^Pal^+^ | | - | 1 | 1.009 | 0.8925 | 1.071 | 0.06789 | Additional File 2: Fig. S1g |
|  | Con siRNA | | + |  |  |  |  |  |  |
|  | TAS1R3 siRNA | | - |  |  |  |  |  |  |
|  | Fru^+^Glu^+^Pal^+^ | | - | 0.5682 | 0.6439 | 0.3649 | 0.6796 | 0.1341 |  |
|  | Con siRNA | | - |  |  |  |  |  |  |
|  | TAS1R3 siRNA | | + |  |  |  |  |  |  |
|  | Fru^+^Glu^+^Pal^+^ | | + | 4.127 | 3.996 | 3.015 | 5.131 | 0.8118 |  |
|  | Con siRNA | | + |  |  |  |  |  |  |
|  | TAS1R3 siRNA | | - |  |  |  |  |  |  |
|  | Fru^+^Glu^+^Pal^+^ | | + | 0.2563 | 0.2652 | 0.1952 | 0.2835 | 0.03505 |  |
|  | Con siRNA | | - |  |  |  |  |  |  |
|  | TAS1R3 siRNA | | + |  |  |  |  |  |  |
| Transcript abundance of TNFa (*TNFa* */GAPDH*) | Fru^+^Glu^+^Pal^+^ | | - | 1 | 1.007 | 0.8302 | 1.154 | 0.1193 | Additional File 2: Fig. S1h |
|  | Con siRNA | | + |  |  |  |  |  |  |
|  | TAS1R3 siRNA | | - |  |  |  |  |  |  |
|  | Fru^+^Glu^+^Pal^+^ | | - | 0.3707 | 0.3754 | 0.323 | 0.4195 | 0.041 |  |
|  | Con siRNA | | - |  |  |  |  |  |  |
|  | TAS1R3 siRNA | | + |  |  |  |  |  |  |
|  | Fru^+^Glu^+^Pal^+^ | | + | 11.86 | 10.27 | 7.956 | 17.57 | 4.171 |  |
|  | Con siRNA | | + |  |  |  |  |  |  |
|  | TAS1R3 siRNA | | - |  |  |  |  |  |  |
|  | Fru^+^Glu^+^Pal^+^ | | + | 1.503 | 1.333 | 1.141 | 2.321 | 0.4697 |  |
|  | Con siRNA | | - |  |  |  |  |  |  |
|  | TAS1R3 siRNA | | + |  |  |  |  |  |  |
| Transcript abundance of GCG (*GCG* */GAPDH*) | Fru^+^Glu^+^Pal^+^ | | - | 0.9827 | 0.9983 | 0.8812 | 1.065 | 0.06656 | Additional File 2: Fig. S1i |
|  | TAS1R3 antagonist (Lactisole) | | - |  |  |  |  |  |  |
|  | Fru^+^Glu^+^Pal^+^ | | - | 0.342 | 0.3407 | 0.2964 | 0.3811 | 0.03427 |  |
|  | TAS1R3 antagonist (Lactisole) | | + |  |  |  |  |  |  |
|  | Fru^+^Glu^+^Pal^+^ | | + | 2.814 | 2.631 | 2.214 | 3.596 | 0.6062 |  |
|  | TAS1R3 antagonist (Lactisole) | | - |  |  |  |  |  |  |
|  | Fru^+^Glu^+^Pal^+^ | | + | 0.3178 | 0.2964 | 0.2731 | 0.3958 | 0.05065 |  |
|  | TAS1R3 antagonist (Lactisole) | | + |  |  |  |  |  |  |
| Transcript abundance of IL1B (*IL1B* */GAPDH*) | Fru^+^Glu^+^Pal^+^ | | - | 1.031 | 1 | 0.8696 | 1.036 | 0.07294 | Additional File 2: Fig. S1j |
|  | TAS1R3 antagonist (Lactisole) | | - |  |  |  |  |  |  |
|  | Fru^+^Glu^+^Pal^+^ | | - | 0.5347 | 0.5326 | 0.2865 | 0.7114 | 0.1555 |  |
|  | TAS1R3 antagonist (Lactisole) | | + |  |  |  |  |  |  |
|  | Fru^+^Glu^+^Pal^+^ | | + | 2.77 | 2.675 | 2.113 | 2.934 | 0.3275 |  |
|  | TAS1R3 antagonist (Lactisole) | | - |  |  |  |  |  |  |
|  | Fru^+^Glu^+^Pal^+^ | | + | 0.5035 | 0.481 | 0.3982 | 0.5681 | 0.07105 |  |
|  | TAS1R3 antagonist (Lactisole) | | + |  |  |  |  |  |  |
| Transcript abundance of IL6 (*IL6 /GAPDH*) | Fru^+^Glu^+^Pal^+^ | | - | 1 | 1.031 | 0.8696 | 1.036 | 0.07294 | Additional File 2: Fig. S1k |
|  | TAS1R3 antagonist (Lactisole) | | - |  |  |  |  |  |  |
|  | Fru^+^Glu^+^Pal^+^ | | - | 0.6416 | 0.6192 | 0.5307 | 0.7722 | 0.1118 |  |
|  | TAS1R3 antagonist (Lactisole) | | + |  |  |  |  |  |  |
|  | Fru^+^Glu^+^Pal^+^ | | + | 2.964 | 2.606 | 1.774 | 4.115 | 0.9795 |  |
|  | TAS1R3 antagonist (Lactisole) | | - |  |  |  |  |  |  |
|  | Fru^+^Glu^+^Pal^+^ | | + | 0.6426 | 0.695 | 0.4114 | 0.8502 | 0.1712 |  |
|  | TAS1R3 antagonist (Lactisole) | | + |  |  |  |  |  |  |
| Transcript abundance of IL8 *(IL8/GAPDH*) | Fru^+^Glu^+^Pal^+^ | | - | 1.293 | 1.237 | 1.133 | 1.583 | 0.1823 | Additional File 2: Fig. S1l |
|  | TAS1R3 antagonist (Lactisole) | | - |  |  |  |  |  |  |
|  | Fru^+^Glu^+^Pal^+^ | | - | 0.4814 | 0.4647 | 0.4453 | 0.5701 | 0.05061 |  |
|  | TAS1R3 antagonist (Lactisole) | | + |  |  |  |  |  |  |
|  | Fru^+^Glu^+^Pal^+^ | | + | 7.651 | 7.345 | 5.391 | 10.27 | 1.885 |  |
|  | TAS1R3 antagonist (Lactisole) | | - |  |  |  |  |  |  |
|  | Fru^+^Glu^+^Pal^+^ | | + | 0.2137 | 0.214 | 0.1228 | 0.3026 | 0.0696 |  |
|  | TAS1R3 antagonist (Lactisole) | | + |  |  |  |  |  |  |
| Transcript abundance of TNFa (*TNFa* */GAPDH*) | Fru^+^Glu^+^Pal^+^ | | - | 1 | 0.9468 | 0.8965 | 1.241 | 0.1395 | Additional File 2: Fig. S1m |
|  | TAS1R3 antagonist (Lactisole) | | - |  |  |  |  |  |  |
|  | Fru^+^Glu^+^Pal^+^ | | - | 0.5446 | 0.5347 | 0.5182 | 0.5973 | 0.03076 |  |
|  | TAS1R3 antagonist (Lactisole) | | + |  |  |  |  |  |  |
|  | Fru^+^Glu^+^Pal^+^ | | + | 4.651 | 4.46 | 2.77 | 7.182 | 1.593 |  |
|  | TAS1R3 antagonist (Lactisole) | | - |  |  |  |  |  |  |
|  | Fru^+^Glu^+^Pal^+^ | | + | 0.4628 | 0.4466 | 0.423 | 0.5483 | 0.04987 |  |
|  | TAS1R3 antagonist (Lactisole) | | + |  |  |  |  |  |  |
| Caloric intake (kcal/24h/mouse) | *Tas1r3*^+/+^ ND | | | 15.27 | 15.36 | 13.01 | 17.79 | 1.36 | Additional File 3: Fig. S2a |
|  | *Tas1r3*^-/-^ ND | | | 12.43 | 12.74 | 10.47 | 15.17 | 1.392 |  |
|  | *Tas1r3*^+/+^ WD | | | 32.08 | 31.33 | 12.63 | 55.1 | 10.04 |  |
|  | *Tas1r3*^-/-^ WD | | | 33.18 | 33.18 | 20.24 | 50.52 | 8.243 |  |
| Diet intake (g/24h/mouse) | *Tas1r3*^+/+^ ND | | | 2.863 | 2.81 | 2.44 | 3.46 | 0.2561 | Additional File 3: Fig. S2b |
|  | *Tas1r3*^-/-^ ND | | | 2.762 | 2.75 | 2.27 | 3.22 | 0.2728 |  |
|  | *Tas1r3*^+/+^ WD | | | 5.008 | 4.326 | 1.24 | 11.23 | 2.444 |  |
|  | *Tas1r3*^-/-^ WD | | | 5.152 | 5.454 | 2.216 | 7.71 | 1.858 |  |
| Water intake (g/24h/mouse) | *Tas1r3*^+/+^ ND | | | 4.957 | 4.82 | 4.4 | 5.53 | 0.4555 | Additional File 3: Fig. S2c |
|  | *Tas1r3*^-/-^ ND | | | 4.182 | 4.03 | 3.63 | 4.94 | 0.4863 |  |
|  | *Tas1r3*^+/+^ WD | | | 8.054 | 8.1 | 7.44 | 8.7 | 0.5027 |  |
|  | *Tas1r3*^-/-^ WD | | | 7.766 | 7.47 | 7.18 | 8.85 | 0.6879 |  |
| Body weight change (% of initial) | *Tas1r3*^+/+^ | day 0 | | 0 | 0 | 0 | 0 | 0 | Additional File 4: Fig. S3a |
|  |  | day 1 | | -0.6967 | -1.02 | -1.19 | 0.12 | 0.7123 |  |
|  |  | day 2 | | -0.2833 | -1.58 | -1.6 | 2.33 | 2.263 |  |
|  |  | day 3 | | -0.87 | -0.89 | -2.31 | 0.59 | 1.45 |  |
|  |  | day 4 | | -0.7467 | -1.55 | -1.83 | 1.14 | 1.64 |  |
|  |  | day 5 | | 0.1933 | 0 | -1.83 | 2.41 | 2.127 |  |
|  |  | day 6 | | 0.82 | 1.29 | -1.99 | 3.16 | 2.607 |  |
|  |  | day 7 | | 0.9567 | 0.19 | -1.08 | 3.76 | 2.509 |  |
|  |  | day 8 | | 0.98 | 0.35 | -0.57 | 3.16 | 1.943 |  |
|  | *Tas1r3*^-/-^ | day 0 | | 0 | 0 | 0 | 0 | 0 |  |
|  |  | day 1 | | 0.01 | -0.075 | -1.54 | 1.54 | 1.121 |  |
|  |  | day 2 | | -0.135 | 0.01 | -2.14 | 1.3 | 1.365 |  |
|  |  | day 3 | | 0.03667 | -0.195 | -1.9 | 1.91 | 1.3 |  |
|  |  | day 4 | | 0.6233 | 0.52 | -0.55 | 1.66 | 0.8804 |  |
|  |  | day 5 | | 1.187 | 1.355 | 0.1 | 1.71 | 0.6017 |  |
|  |  | day 6 | | 0.9083 | 0.935 | -0.45 | 1.99 | 0.8397 |  |
|  |  | day 7 | | 0.91 | 0.945 | -0.69 | 2.17 | 0.9674 |  |
|  |  | day 8 | | 0.8233 | 0.955 | -0.98 | 2.6 | 1.318 |  |
|  | *Tas1r3*^+/+^ + DSS | day 0 | | 0 | 0 | 0 | 0 | 0 |  |
|  |  | day 1 | | 0.7167 | 1.005 | -1.94 | 3.81 | 2.324 |  |
|  |  | day 2 | | 0.055 | -0.1 | -2.37 | 4.34 | 2.554 |  |
|  |  | day 3 | | -0.3883 | -1.02 | -2.79 | 4.87 | 2.764 |  |
|  |  | day 4 | | 0.06667 | -0.345 | -2.62 | 4.26 | 2.28 |  |
|  |  | day 5 | | -6.888 | -6.375 | -12.2 | -2.7 | 3.813 |  |
|  |  | day 6 | | -15.37 | -14.18 | -20.85 | -10.12 | 4.308 |  |
|  |  | day 7 | | -22.93 | -22.08 | -29.85 | -17.28 | 5.268 |  |
|  |  | day 8 | | -32.21 | -29.03 | -41.4 | -25.72 | 6.901 |  |
|  | *Tas1r3*^-/-^ + DSS | day 0 | | 0 | 0 | 0 | 0 | 0 |  |
|  |  | day 1 | | 0.404 | 0.18 | -0.81 | 1.92 | 1.239 |  |
|  |  | day 2 | | 0.818 | 0.75 | -0.16 | 1.64 | 0.7039 |  |
|  |  | day 3 | | 1.222 | 1.35 | -0.49 | 2.31 | 1.048 |  |
|  |  | day 4 | | 0.818 | 1.88 | -3.73 | 2.96 | 2.705 |  |
|  |  | day 5 | | -2.368 | -2.28 | -6.2 | 1.41 | 2.709 |  |
|  |  | day 6 | | -8.616 | -9.81 | -13.24 | -3.79 | 3.935 |  |
|  |  | day 7 | | -14.11 | -13.61 | -18.24 | -10.98 | 2.827 |  |
|  |  | day 8 | | -17.82 | -18.59 | -19.96 | -15.36 | 1.886 |  |
| Disease activity index (DAI) score | *Tas1r3*^+/+^ | day 0 | | 0 | 0 | 0 | 0 | 0 | Additional File 4: Fig. S3b |
|  |  | day 1 | | 0 | 0 | 0 | 0 | 0 |  |
|  |  | day 2 | | 0 | 0 | 0 | 0 | 0 |  |
|  |  | day 3 | | 0 | 0 | 0 | 0 | 0 |  |
|  |  | day 4 | | 0 | 0 | 0 | 0 | 0 |  |
|  |  | day 5 | | 0 | 0 | 0 | 0 | 0 |  |
|  |  | day 6 | | 0 | 0 | 0 | 0 | 0 |  |
|  |  | day 7 | | 0 | 0 | 0 | 0 | 0 |  |
|  |  | day 8 | | 0 | 0 | 0 | 0 | 0 |  |
|  | *Tas1r3*^-/-^ | day 0 | | 0 | 0 | 0 | 0 | 0 |  |
|  |  | day 1 | | 0 | 0 | 0 | 0 | 0 |  |
|  |  | day 2 | | 0 | 0 | 0 | 0 | 0 |  |
|  |  | day 3 | | 0 | 0 | 0 | 0 | 0 |  |
|  |  | day 4 | | 0 | 0 | 0 | 0 | 0 |  |
|  |  | day 5 | | 0 | 0 | 0 | 0 | 0 |  |
|  |  | day 6 | | 0 | 0 | 0 | 0 | 0 |  |
|  |  | day 7 | | 0 | 0 | 0 | 0 | 0 |  |
|  |  | day 8 | | 0 | 0 | 0 | 0 | 0 |  |
|  | *Tas1r3*^+/+^ + DSS | day 0 | | 0 | 0 | 0 | 0 | 0 |  |
|  |  | day 1 | | 0 | 0 | 0 | 0 | 0 |  |
|  |  | day 2 | | 0 | 0 | 0 | 0 | 0 |  |
|  |  | day 3 | | 3.222 | 2 | 2 | 6 | 1.716 |  |
|  |  | day 4 | | 4.444 | 4 | 2 | 8 | 1.944 |  |
|  |  | day 5 | | 6 | 6 | 5 | 8 | 1.118 |  |
|  |  | day 6 | | 8.556 | 9 | 7 | 10 | 0.8819 |  |
|  |  | day 7 | | 11.44 | 11 | 11 | 12 | 0.527 |  |
|  |  | day 8 | | 11.89 | 12 | 11 | 12 | 0.3333 |  |
|  | *Tas1r3*^-/-^ + DSS | day 0 | | 0 | 0 | 0 | 0 | 0 |  |
|  |  | day 1 | | 0 | 0 | 0 | 0 | 0 |  |
|  |  | day 2 | | 0 | 0 | 0 | 0 | 0 |  |
|  |  | day 3 | | 2.429 | 2 | 2 | 3 | 0.5345 |  |
|  |  | day 4 | | 3.857 | 4 | 2 | 6 | 1.215 |  |
|  |  | day 5 | | 3.857 | 4 | 2 | 6 | 1.215 |  |
|  |  | day 6 | | 5 | 4 | 4 | 9 | 1.915 |  |
|  |  | day 7 | | 5.571 | 5 | 3 | 9 | 1.988 |  |
|  |  | day 8 | | 5.857 | 6 | 4 | 8 | 1.464 |  |
| Colon length (cm) | *Tas1r3*^+/+^ | | | 7.767 | 7.8 | 7.5 | 8 | 0.2517 | Additional File 4: Fig. S3c |
|  | *Tas1r3*^-/-^ | | | 8.017 | 8.25 | 6 | 9 | 1.072 |  |
|  | *Tas1r3*^+/+^ + DSS | | | 4.188 | 4.1 | 3.4 | 5.3 | 0.6151 |  |
|  | *Tas1r3*^-/-^ + DSS | | | 5.757 | 5.7 | 5 | 6.5 | 0.574 |  |
| Small intestine inflammation score | *Tas1r3*^+/+^ | | | 0 | 0 | 0 | 0 | 0 | Additional File 4: Fig. S3d |
|  | *Tas1r3*^-/-^ | | | 0 | 0 | 0 | 0 | 0 |  |
|  | *Tas1r3*^+/+^ + DSS | | | 8.5 | 8 | 7.5 | 10.5 | 1 |  |
|  | *Tas1r3*^-/-^ + DSS | | | 1.429 | 0 | 0 | 6 | 2.507 |  |
| Colon inflammation score | *Tas1r3*^+/+^ | | | 0 | 0 | 0 | 0 | 0 | Additional File 4: Fig. S3e |
|  | *Tas1r3*^-/-^ | | | 0 | 0 | 0 | 0 | 0 |  |
|  | *Tas1r3*^+/+^ + DSS | | | 10.7 | 11 | 10 | 11 | 0.4472 |  |
|  | *Tas1r3*^-/-^ + DSS | | | 4.6 | 4 | 4 | 6 | 0.8944 |  |
| Small intestine CD45^+^ inflammation score | *Tas1r3*^+/+^ | | | 2.431 | 1.562 | 0.065 | 8.992 | 3.267 | Additional File 4: Fig. S3f |
|  | *Tas1r3*^-/-^ | | | 2.194 | 1.41 | 0.114 | 4.321 | 1.734 |  |
|  | *Tas1r3*^+/+^ + DSS | | | 20.58 | 22.44 | 14.83 | 25.33 | 4.105 |  |
|  | *Tas1r3*^-/-^ + DSS | | | 8.425 | 7.669 | 2.321 | 14.48 | 4.429 |  |
| Colon CD45^+^ inflammation score | *Tas1r3*^+/+^ | | | 3.574 | 3.842 | 1.162 | 5.992 | 1.722 | Additional File 4: Fig. S3g |
|  | *Tas1r3*^-/-^ | | | 3.48 | 3.472 | 2.008 | 5.114 | 1.118 |  |
|  | *Tas1r3*^+/+^ + DSS | | | 38.08 | 36.29 | 33.38 | 43.65 | 4.087 |  |
|  | *Tas1r3*^-/-^ + DSS | | | 15.42 | 14.48 | 5.49 | 27.32 | 8.055 |  |
