# Supplementary material for: Gut taste receptor type 1 member 3 is an intrinsic regulator of Western diet-induced intestinal inflammation
Source: BMC Med. 2023 Apr 28;21:165. doi: 10.1186/s12916-023-02848-0 (PMC10148556; doi:10.1186/s12916-023-02848-0)
Supplement: Supplementary file 2 — Additional file 2: Figure S1. The effect of inhibition of TAS1R3 by siRNA transfection and antagonist on pro-inflammatory cytokine expression in EECs. [file 12916_2023_2848_MOESM2_ESM.docx]

**
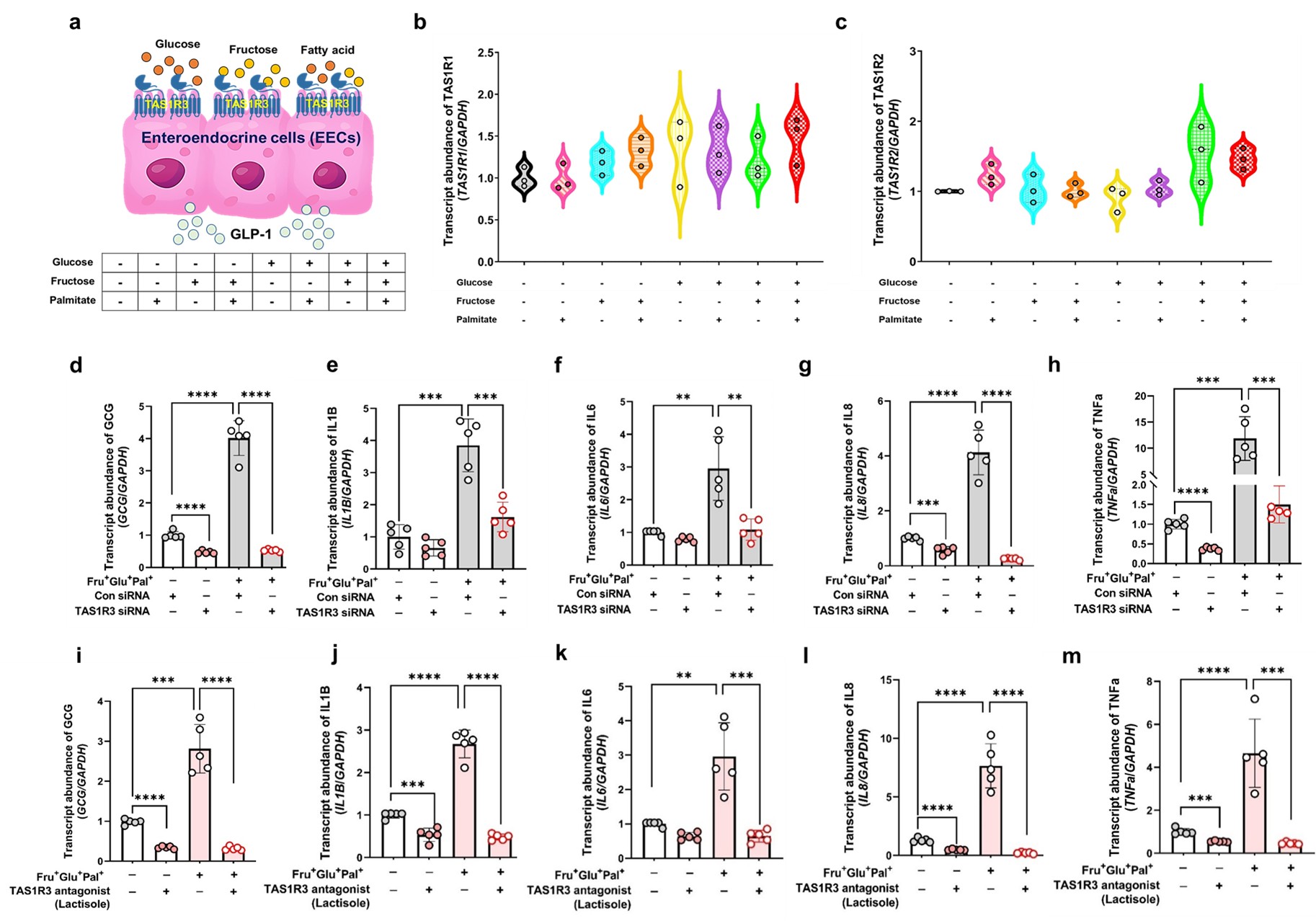
**

**Fig. S1. Inhibition of TAS1R3 by siRNA transfection and antagonist downregulates pro-inflammatory cytokine expression in EECs. (a)** Schematic depicting how TAS1R3 may regulate GLP-1 secretion with nutrient ligand stimuli in EECs. **(b)** and **(c)** qRT-PCR analysis of relative intestinal mRNA expression of *TAS1R1* and *TAS1R2* in NCI-H716 cells after 12 h of treatment with individual or combined glucose, fructose, and palmitate to activate their receptor. **(d–h)** NCI-H716 cells were transfected with TAS1R3 (10 nM) or scrambled control (10 nM) siRNA for 48 h and then stimulated with or without fructose (10 mM), glucose (10 mM), and palmitate (10 μM) for 12 h. mRNA expression of **(d)** *GLP1*, **(e)** *IL1B*, **(f)** *IL6*, **(g)** *IL8*, and **(h)** *TNFɑ* in EECs was measured by qRT-PCR. **(i–m)** NCI-H716 cells were pretreated with the TAS1R3 antagonist, lactisole (2.5 mM), for 30 min and then stimulated with or without fructose (10 mM), glucose (10 mM), and palmitate (10 μM) for 12 h. mRNA expression of **(i)** *GLP1*, **(j)** *IL1B*, **(k)** *IL6*, **(l)** *IL8*, and **(m)** *TNFɑ* in EECs was measured using qRT-PCR. Values represent means ± standard errors of the mean. **P* < 0.05, ***P* < 0.01, ****P* < 0.001, and *****P* < 0.0001 (analysis of variance followed by Bonferroni post-hoc test). EEC, enteroendocrine cell; qRT-PCR, quantitative reverse transcription PCR.
