# Supplementary material for: Gut taste receptor type 1 member 3 is an intrinsic regulator of Western diet-induced intestinal inflammation
Source: BMC Med. 2023 Apr 28;21:165. doi: 10.1186/s12916-023-02848-0 (PMC10148556; doi:10.1186/s12916-023-02848-0)
Supplement: Supplementary file 3 — Additional file 3: Figure S2. Comparison of caloric intake of Tas1r3−/− and Tas1r3+/+ mice fed WD or ND for 10 weeks ad libitum. [file 12916_2023_2848_MOESM3_ESM.docx]

**
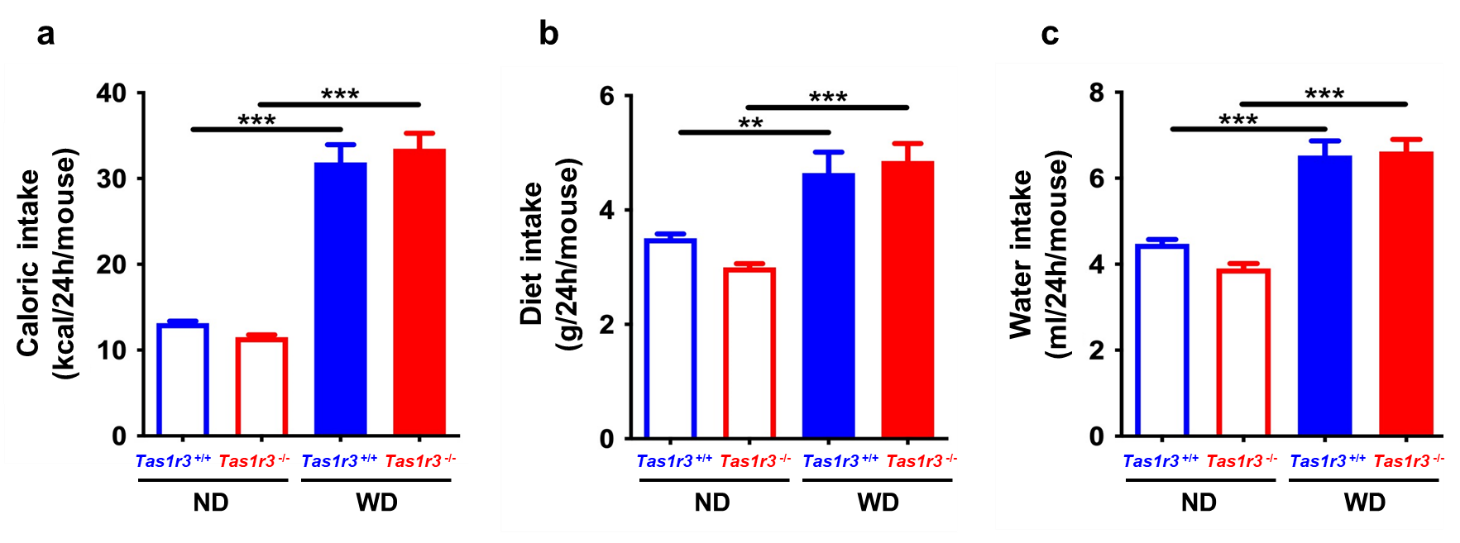
**

**Fig. S2: Caloric intake of *Tas1r3*^−/−^ and *Tas1r3*^+/+^ mice fed WD or ND for 10 weeks *ad libitum*.**

**(a)** Calorie intake, **(b)** dietary intake, and **(c)** water intake in *Tas1r3*^−/−^ and *Tas1r3*^+/+^ mice fed WD or ND (n = 10 mice/group). Values represent means ± standard errors of the mean. **P* < 0.05, ***P* < 0.01, ****P* < 0.001, and *****P* < 0.0001 (analysis of variance followed by Bonferroni post-hoc test). ND, normal diet; WD, Western diet.
