# Supplementary material for: Gut taste receptor type 1 member 3 is an intrinsic regulator of Western diet-induced intestinal inflammation
Source: BMC Med. 2023 Apr 28;21:165. doi: 10.1186/s12916-023-02848-0 (PMC10148556; doi:10.1186/s12916-023-02848-0)
Supplement: Supplementary file 4 — Additional file 4: Figure S3. The effect of TAS1R3 deficiency on intestinal inflammation in a murine DSS-colitis model. [file 12916_2023_2848_MOESM4_ESM.docx]

**
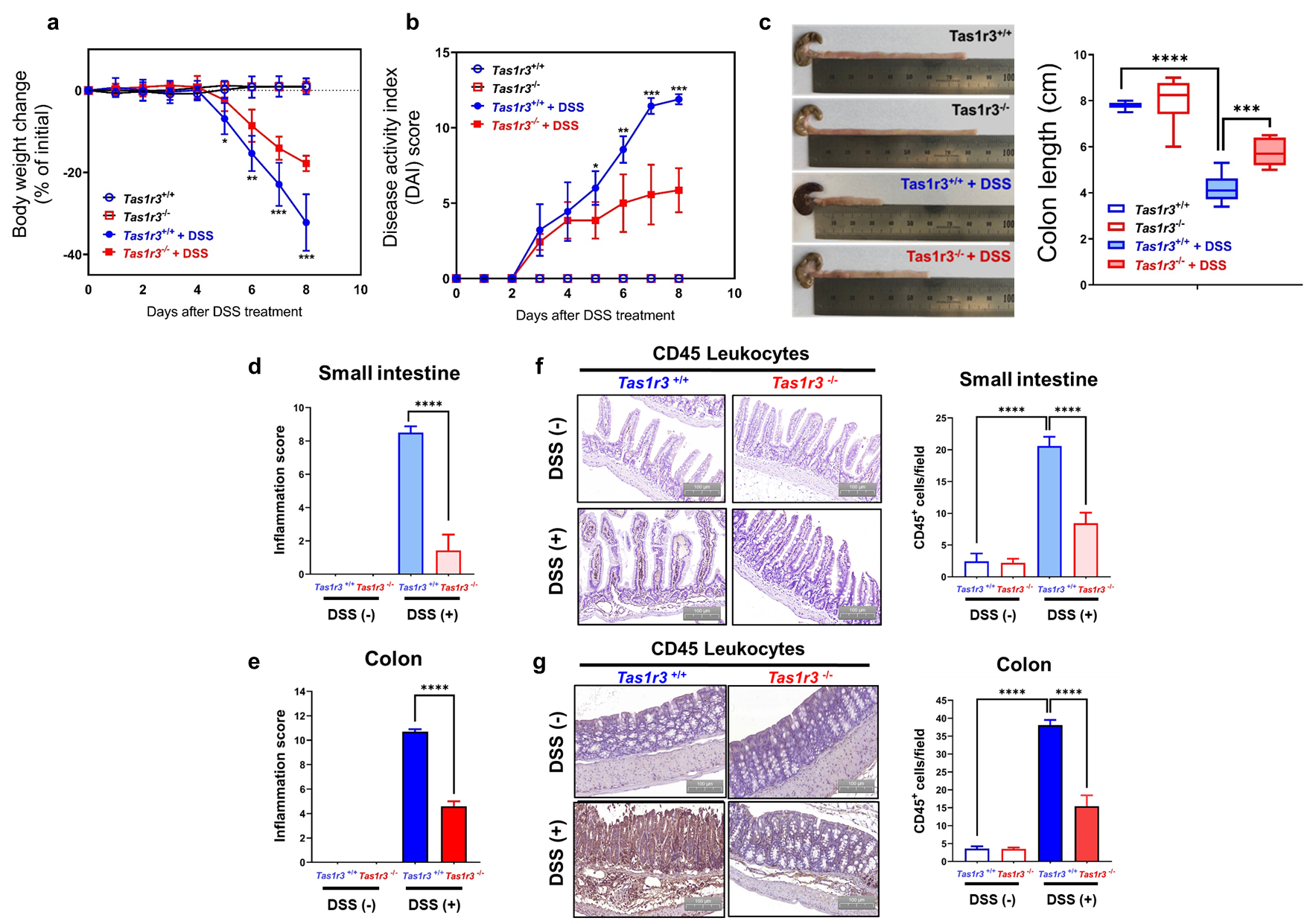
**

**Fig. S3: TAS1R3 deficiency ameliorates intestinal inflammation in a murine DSS-colitis model.**

Wild-type and *Tas1r3*-knockout mice were administered 2% DSS in their drinking water for 7 days. **(a)** Percentage body weight change (n = 7 mice/group). **(b)** Colitis disease activity index based on the severity of diarrhea and rectal bleeding (n = 7 mice/group). **(c)** Representative colon lengths of wild-type and *Tas1r3-*knockout mice 7 days after DSS administration. **(d–e)** Inflammation scores based on histological staining of small and large intestinal tissue from wild-type and *Tas1r3-*knockout mice 7 days after DSS administration (n = 7 mice/group). **(f)** and **(g)** CD45^+^ leukocyte infiltration in the small and large intestine of DSS-treated mice. Immunohistochemistry was performed using an antibody specific for CD45 leukocytes (n = 7 mice/group). Data are expressed as means ± standard errors of the mean. *P < 0.05, **P < 0.01, ***P < 0.001, and ****P < 0.0001 (unpaired Student’s *t*-test). DSS, dextran sulfate sodium.
