# Supplementary material for: Gut taste receptor type 1 member 3 is an intrinsic regulator of Western diet-induced intestinal inflammation
Source: BMC Med. 2023 Apr 28;21:165. doi: 10.1186/s12916-023-02848-0 (PMC10148556; doi:10.1186/s12916-023-02848-0)
Supplement: Supplementary file 5 — Additional file 5: Figure S4. Schematic depicting the roles of TAS1R3 in the intestinal inflammation. [file 12916_2023_2848_MOESM5_ESM.docx]

**
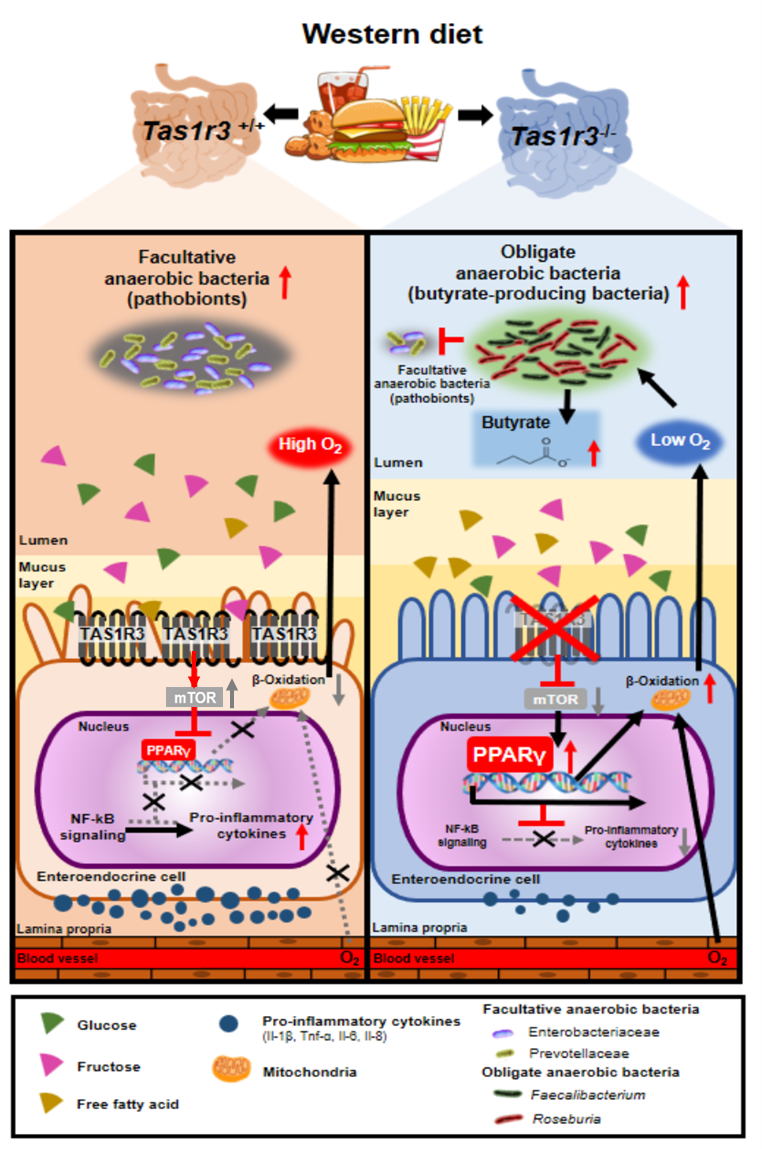
**

**Fig. S4. Schematic depicting that nutrient-sensing taste receptor TAS1R3 regulates intestinal inflammation via the mTOR-PPARγ axis.** Enteroendocrine cells harboring TAS1R3 secrete inflammatory cytokines following direct activation by Western diet (WD) stimuli. Despite prolonged ingestion of WD, *Tas1r3*-deficient mice were protected from intestinal inflammation. Mechanistically, TAS1R3 deficiency suppresses the mTOR signaling pathway, resulting in increased expression of intestinal PPAR-γ. Elevated PPARγ expression induced by TAS1R3 deficiency may protect against intestinal inflammation through two main pathways: (1) inhibiting the production of proinflammatory cytokines through its action on kinases and transcription factors, such as NF-κB, and (2) driving β-oxidation in the intestine, leading to expansion of obligate anaerobic butyrate-producing bacteria by maintaining a hypoxic state in the gut, with these obligated bacteria suppressing the dysbiotic expansion of facultative anaerobic pathogenic bacteria by competing for oxygen.
