# Supplementary figures and images for: Gut taste receptor type 1 member 3 is an intrinsic regulator of Western diet-induced intestinal inflammation
Source: BMC Med. 2023 Apr 28;21:165. doi: 10.1186/s12916-023-02848-0 (PMC10148556; doi:10.1186/s12916-023-02848-0)

**
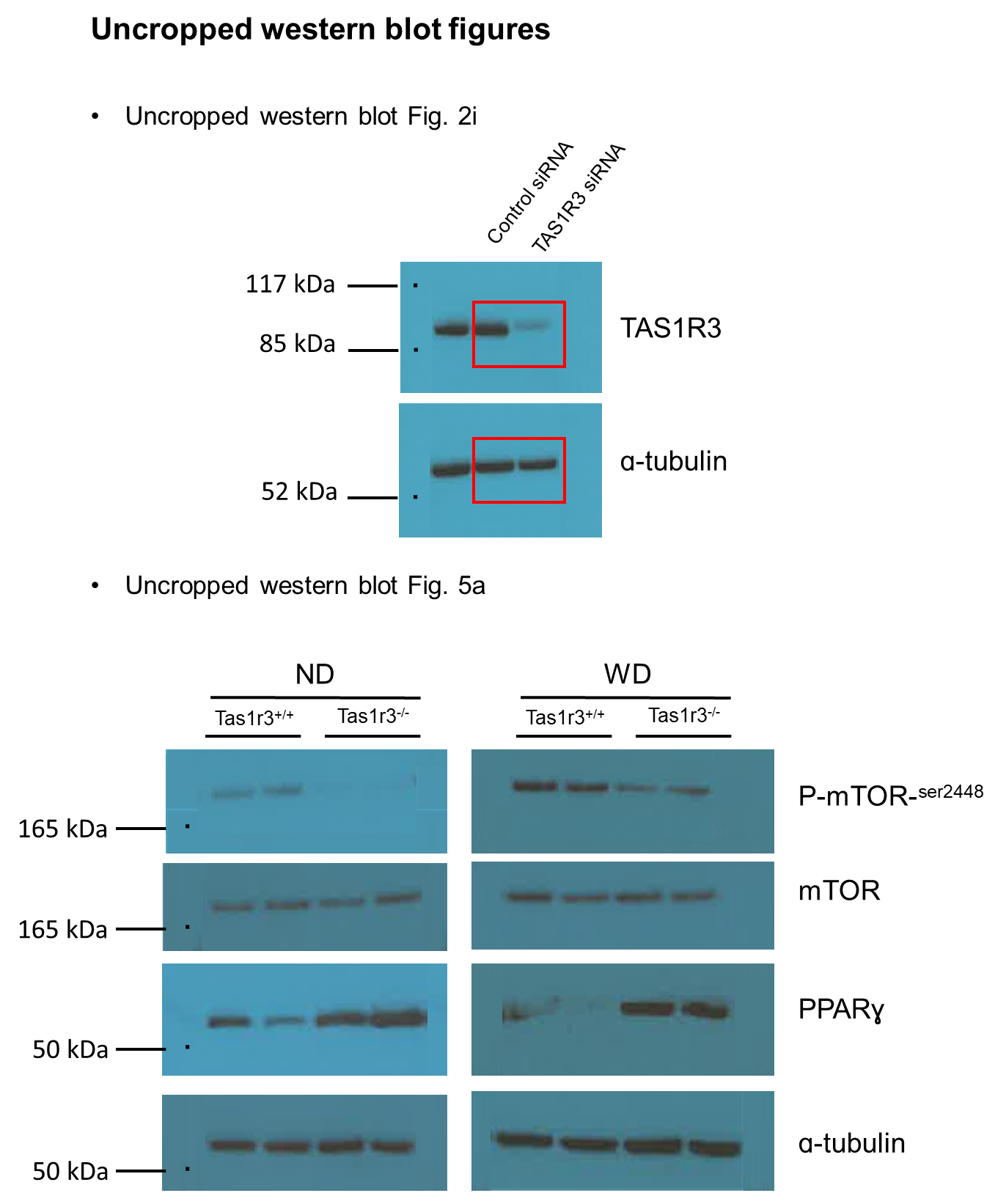
**

**
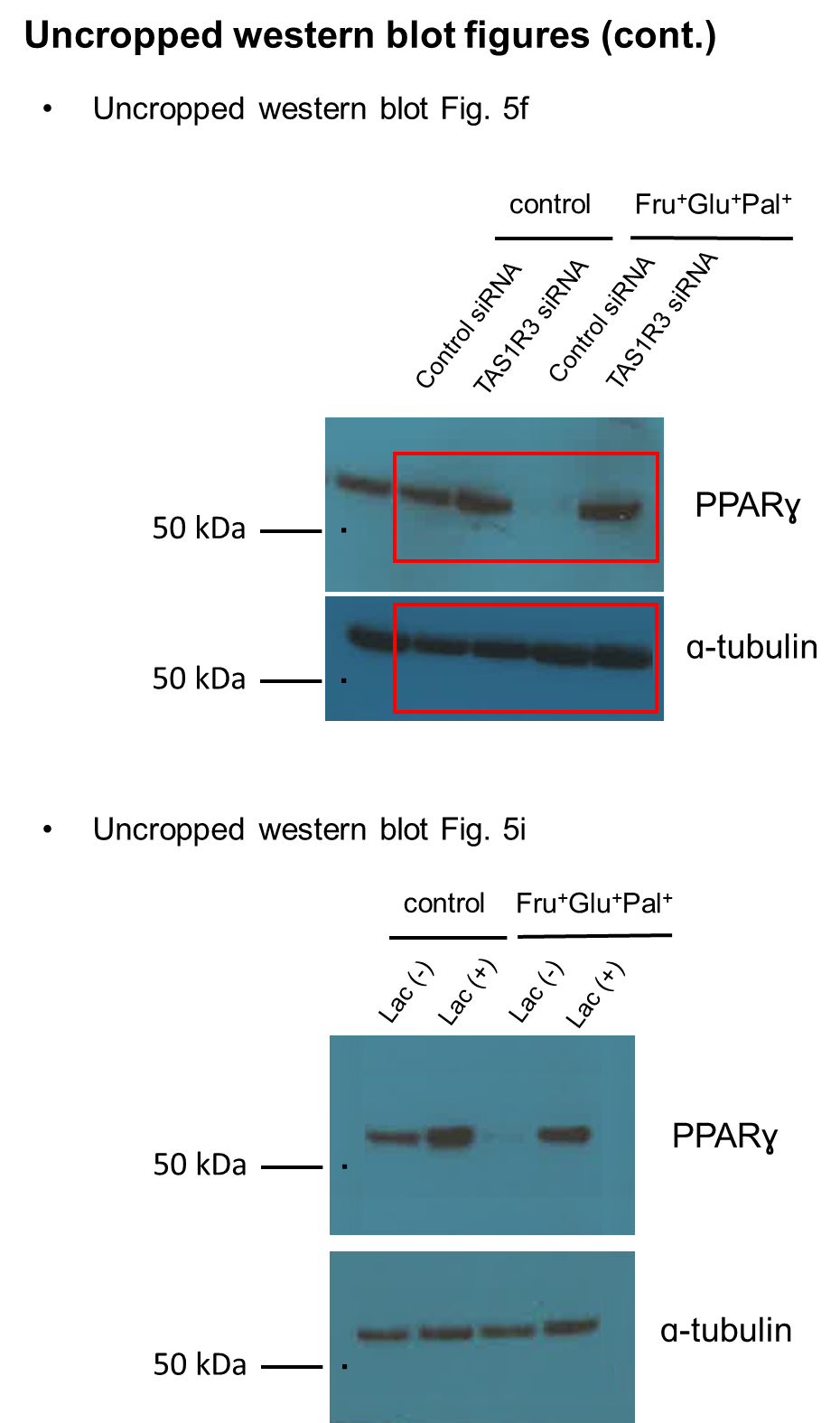
**

**
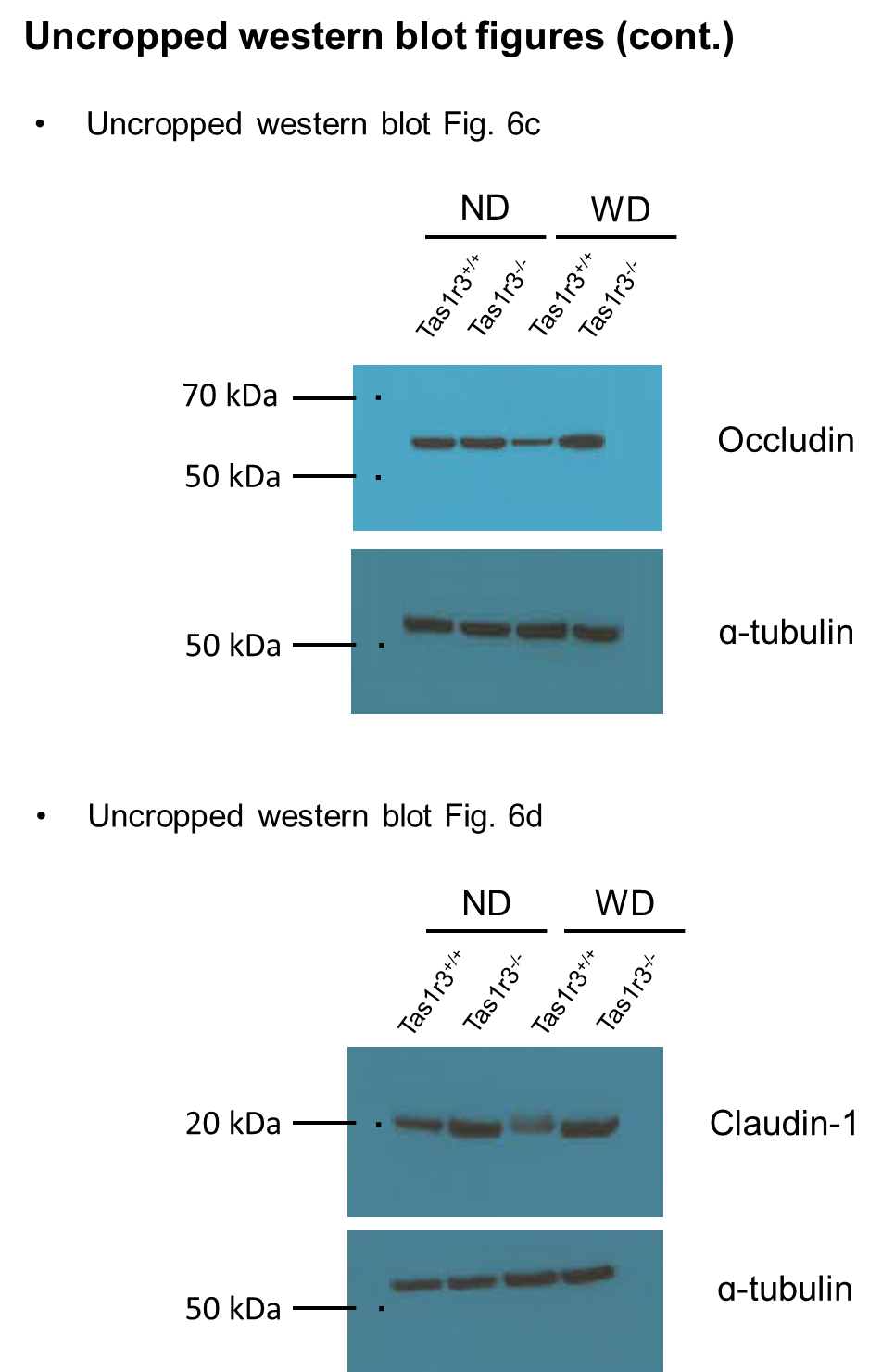
**

Supplement: Supplementary file 6 — Additional file 6. Uncropped western blot figures. [file 12916_2023_2848_MOESM6_ESM.docx]
